# DSM: Deep sequential model for complete neuronal morphology representation and feature extraction

**DOI:** 10.1016/j.patter.2023.100896

**Published:** 2023-12-13

**Authors:** Feng Xiong, Peng Xie, Zuohan Zhao, Yiwei Li, Sujun Zhao, Linus Manubens-Gil, Lijuan Liu, Hanchuan Peng

**Affiliations:** 1 New Cornerstone Science Laboratory, SEU-ALLEN Joint Center, Institute for Brain and Intelligence, Southeast University, Nanjing, Jiangsu 210096, China; 2 School of Biological Science and Medical Engineering, Southeast University, Nanjing, Jiangsu 210096, China; 3 School of Computer Science and Engineering, Southeast University, Nanjing, Jiangsu 210096, China

**Keywords:** mouse neuron, brain regions, morphological classification, deep learning

## Abstract

The full morphology of single neurons is indispensable for understanding cell types, the basic building blocks in brains. Projecting trajectories are critical to extracting biologically relevant information from neuron morphologies, as they provide valuable information for both connectivity and cell identity. We developed an artificial intelligence method, deep sequential model (DSM), to extract concise, cell-type-defining features from projections across brain regions. DSM achieves more than 90% accuracy in classifying 12 major neuron projection types without compromising performance when spatial noise is present. Such remarkable robustness enabled us to efficiently manage and analyze several major full-morphology data sources, showcasing how characteristic long projections can define cell identities. We also succeeded in applying our model to both discovering previously unknown neuron subtypes and analyzing exceptional co-expressed genes involved in neuron projection circuits.

## Highlights

• We developed deep models for characterizing complete single-neuron morphology
• Long projections of neurons are critical features for identifying cell types
• Our models can classify neuron morphologies with remarkable robustness and efficiency

## The bigger picture

Delineating the cell atlas of the brain is key to understanding intelligence. The complex and exquisite morphological patterns of neuronal cells provide structural support for signal transmission in our brains. Over the past years, a significant body of neuronal morphology data has been accumulated, raising urgent needs for computational tools to fully extract features relevant to cellular identity and connectivity. Here, we find that the long-projecting trajectory of a neuron, which has been overseen by existing algorithms, is an effective feature for cell-type definition. We utilize a series of natural language models to decipher the projection pathways and significantly improve the accuracy of neuronal type classification. Our work provides a novel quantitative strategy for creating a consensus of a neuronal cell atlas, which is potentially applicable to understanding defects in neurological diseases.

## Teaser

Reliable and efficient characterization of neuron morphology is crucial for determining neuron cell types, which is a key question for understanding the complex atlas of the brain. This article presents a deep learning method, deep sequential model (DSM), to characterize complete neuron morphology. The DSM model organizes neuronal topology as sequence representation and extracts defining features from neuronal projections across brain regions. The results demonstrate the performance and generalizability of DSM on multiple datasets.

## Introduction

The classification of neuronal types is crucial to understanding the complex circuits of the brain. Neuronal classification requires comprehensive characterization at the levels including morphology, electrical properties, transcriptomics, or a combination of them.^1^^,^^2^ Among these, neuron morphology provides key implications for cellular identity and neuronal connectivity. However, morphological studies have long been restricted to the soma-proximal areas, being limited by the existing imaging technologies. Recently, large-scale labeling, imaging, and reconstruction technologies have enabled the characterization of complete neuron morphologies at the whole-brain level.^3^^,^^4^ These studies showed cell-type diversity and sub-types observed in whole brain level provide new clues for understanding neuronal circuits.^4^

To describe complex neuron morphologies and to classify cell types, two key questions need to be addressed: feature extraction and quantitative comparison. Several feature extraction methods have been proposed, including vertex analysis,^5^ fan-in analysis,^6^ fractal analysis,^7^ and L-Measure morphometrics.^8^^,^^9^ The spatial distribution of dendritic arbors has also been demonstrated to be a relevant feature.^10^ For quantitative comparison of morphological data, both supervised and unsupervised algorithms have been proposed.^11^^,^^12^ However, these approaches are designed for analyzing dendrites and local axons arbors and the extracted features may not be suitable for characterization of morphologies with long-range projections.

In recent years, several methods have been proposed for studying the full morphology of projecting neurons. Considering the spatial distribution of both local and distal neuronal branches, NBLAST was designed by Costa et al.^13^ for measuring pairwise neuronal similarity. Integrating topological branching patterns with spatial information, persistent homology was introduced to compare neuron structures and classify a large collection of neuronal structures.^14^^,^^15^ BlastNeuron compares morphological similarities using a structural alignment approach.^16^ However, the accuracy and robustness of such approaches are considerably affected by within-type diversity and registration precision.

The long-range projection path is a biologically relevant feature for defining cell types,^4^ which is neglected by the abovementioned approaches. Here, we propose a strategy to encode the projection path, a tree-like structure of 3D coordinates in the brain space, as a computable characteristic for quantitative analysis. We organized the neuronal tree topology as a sequence representation, which was used for identification of structural motifs.^17^^,^^18^ The sequence structure provides a natural representation of the projection orders and allows for the application of a series of deep sequential models (DSMs). For cell-type classification tasks, we implemented and trained a hierarchical attention network^19^ model (DSM-HAN) and demonstrated its outstanding performance. For the measurement of cell-cell similarity, we trained a sequential autoencoder model (DSM-AE) to give each cell a concise representation, which encoded information of both projection strength and order of innervated brain regions. We showed the usage of DSM-AE in unsupervised clustering and automated cell-type annotation of large datasets. With DSM-AE feature encodings, we built a database and provided an online service for fast retrieval of neuron morphologies and cell-type annotation.

## Results

### Overview of the model structure, datasets, and applications

**Figure 1:**
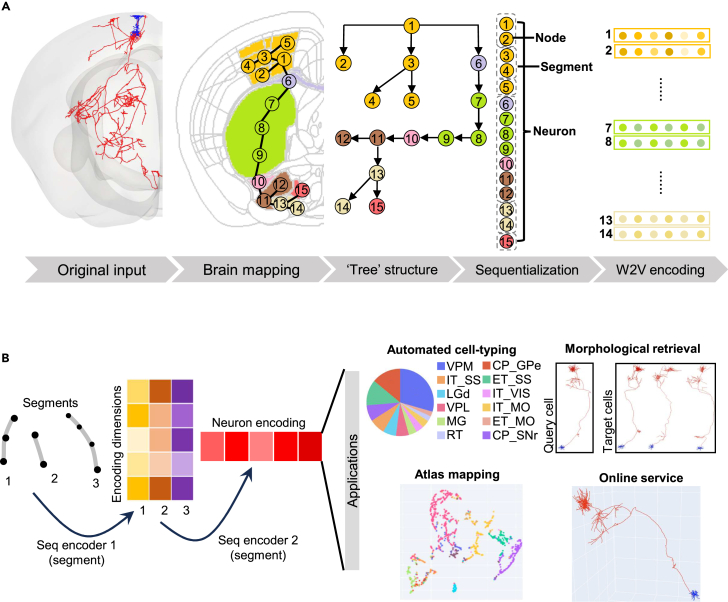
Overview of the sequential model and its applications (A) Raw data transformation process including mapping the nodes of reconstructed neurons to brain regions, tree structure representation, depth-first search, which disassembles the tree structure as segment sequences, and word2vec (W2V) encoding that represents each node as the embedding of its brain region. (B) Neuron encoding process. A neuron is encoded by two steps of recursive neural network transformation. This process encodes neurons with variable sizes as a vector of the same dimensions, which is then used as input for automated cell-type classification, unsupervised atlas mapping, and morphological retrieval.

To extract features and characterize neuronal morphologies, we constructed several DSMs, serving for multiple applications (Figure 1). We built a pipeline for preprocessing and feature extraction for neuron reconstruction (Figure 1A). The original input is the digital reconstruction of neuronal morphology, including the complete long-range projection. Reconstructed neurons were registered to CCF reference space^20^ after which each segment of the reconstruction belongs to a certain brain region according to its 3D location in the space. We applied a depth-first traversal algorithm to convert the tree structure of the morphologically reconstructed neurons into a sequence of brain regions (see experimental procedures), where each node in the sequence is represented as a one-hot encoding. The designed traversal order makes child branches close to their parent branches, allowing for more compact sequentialization of the local morphologies along the projection path. Each node is assigned to one of 316 manually curated non-overlapping brain areas^21^ with highly variable spatial proximity and functional properties.

To reduce redundancy of the one-hot encoding vectors, we introduced the word2vec (W2V) module^22^ (see experimental procedures) for feature compression and extraction. We applied this model to over 1,000 neurons of multiple types and found that W2V is not only able to convert the 316-dimension vectors into dense vectors, but also enhances the robustness of feature representation. Thus, we used the W2V module to generate the input for both supervised classification and unsupervised representation (Figure 1B).

The DSM consists of two downstream models, DSM-HAN and DSM-AE, performing supervised classification and unsupervised representation separately. In the supervised module, a hierarchical attention network is adopted as DSM-HAN for cell-type classification. DSM-HAN reviews neuron morphologies in a hierarchical manner including node level, segment level, and the neuron level, providing both segment-level encodings and neuron level encodings. The model shows its interpretability by encoding neighboring segments into similar vectors (Figure S1). We trained the DSM-HAN model with 1,282 neurons that belong to 12 cell types (caudate putamen substantia nigra pars reticulate [CP_SNr], caudate putamen globus pallidus externa [CP_GPe], ventral posteromedial nucleus of the thalamus [VPM], extratelencephalic somatosensory cortex [ET_SS], intratelencephalic somatosensory cortex [IT_SS], ventral posterolatral nucleus of the thalamus [VPL], dorsal part of the lateral geniculate complex [LGd], medial geniculate complex [MG], intratelencephalic visual area [IT_VIS], intratelencephalic motor cortex [IT_MO], thalamic reticular nucleus [RT], and extratelencephalic motor cortex [ET_MO]) from a dataset recently obtained by our team.^4^

In the unsupervised module, we trained a DSM-AE model for data exploration, including unsupervised clustering and retrieval of similar morphologies. Details of the W2V model and DSM are introduced in the following sections.

### W2V: Distributed vectors of brain regions

In this study, we propose a strategy to utilize brain regions, to help identify the cell types. Sub-regions within one major brain region, such as the somatosensory cortex, may share functional, spatial, and developmental similarity. Thus, neurons projecting to these closely located sub-regions might belong to the same cell type (e.g., VPM neurons may project SSp-m or SSp-bfd).

To determine the projection regions, the registration of neuron reconstructions from different brains can also introduce certain levels of error. In addition, current characterization for brain regions is not only lack of connectivity profiles, but also cannot reach the single-cell resolution. We can address these concerns by proposing the feature, our brain region encodings. The feature is produced by the W2V algorithm, which is a classic method for feature extraction and compression in NLP tasks.^22^ Since our neuron morphology is converted into sequences, it provided the facility to embed nodes (brain regions) from the sequences. W2V assigns each node with a unique encoding (fixed-length vectors) indicating its semantics such that nodes with similar semantics or synonymous have closer encodings. By applying W2V, we were able to encode brain regions as dense vectors, reflecting their functional and spatial similarity, which benefits the downstream analysis.

**Figure 2:**
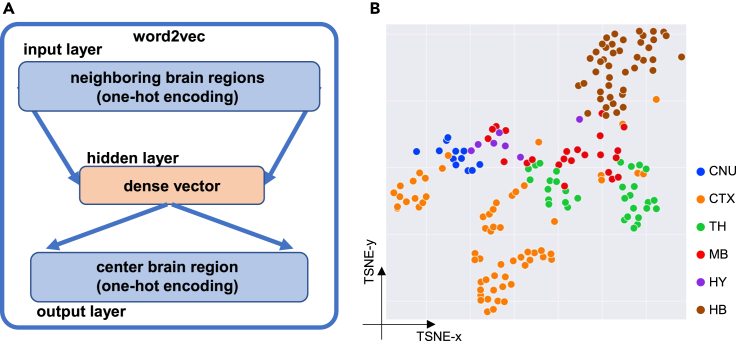
Word2vec: Distributed vectors of brain regions (A) Word2vec network structure. (B) Distribution of trained brain regions in a t-SNE layout. To display the training result, sub-regions are separated into six major regions: CNU (cerebral nuclei), CTX (cerebral cortex), TH (thalamus), MB (midbrain), HY (hypothalamus), and HB (hindbrain). Structures belonging to the same major region are displayed in the same color. Each dot represents a sub-region reduced from W2V encodings, and the colors indicate the major brain regions.

Our W2V neural network is composed of three layers, including an input layer, a hidden layer, and an output layer, as shown in Figure 2A. The input and output data are one-hot encodings of brain regions. Fed with the sub-sequence of context nodes as input, the model predicts the one-hot encoding of its center node. The dense encoding of center node comes from the average hidden layer representation of input nodes (see Table S1 for model parameters; see experimental procedures). The training process minimizes the prediction error of the center node.

To enhance the robustness of the W2V, we generated an augmented dataset by introducing Gaussian noise to node coordinates and used it as the training data of W2V (see experimental procedures). The augmented dataset contains 316 brain regions and 5,370 cells. These brain region embeddings can be grouped as 6 major brain regions: cerebral nuclei, cerebral cortex, thalamus, midbrain, hypothalamus, and hindbrain. After training, each brain region was encoded as a 6-dimension embedding vector (Table S2). The dimension of W2V encoding was chosen by the least training loss of W2V (Figure S2A). Dimension reduction (t-SNE) of these W2V encodings shows that the 316 sub-regions within the same major brain regions are clustered together (Figure 2B).

### HAN: Supervised cell-type classification

**Figure 3:**
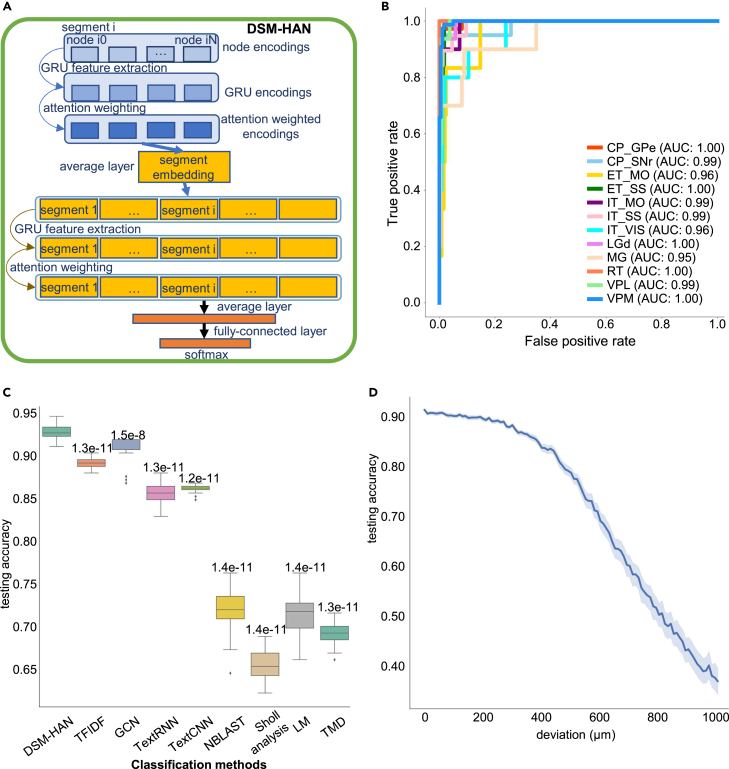
Hierarchical attention network: Supervised cell-type classification (A) Hierarchical attention network structure. Blue blocks: node-level neural networks. Yellow blocks: sentence-level neural networks. Orange blocks: neuron-level neural networks. (B) The receiver operating characteristic (ROC) curve and area under the curve of the DSM-HAN classifier. Taking multi-class classification as 12 binary classifications, we calculated false positive rate and true positive rate for each binary classification and plot the ROC curves. The ROC curve of each type is displayed in one color. (C) Comparison between methods. Each method was tested by 30 times of cross-validation, and results are displayed by boxplots (numbers above box: p values of the Mann-Whitney U rank test (one-side) on test accuracy between DSM-HAN and others). (D) Robustness test: the testing accuracy for noise levels ranging from 10 to 1,000 μm. The accuracy curve is aggregated over repeated values (each noise level with 30 independent trainings and testings), showing the mean testing accuracy and 95% confidence interval.

For supervised morphological classification, we adopted a DSM-HAN model, which was originally used for document classification.^19^ The intuition is to treat a neuron sequence (a sequence of W2V-encoded brain regions) as a document (a sequence of words), and treat neuron segments (sub-sequences) as sentences. The DSM-HAN architecture reviews the sequence and extracts features following the order of nodes to segments and segments to neurons. W2V encodings of nodes in the same segment are seen as independent sub-sequences, representing local morphologies. The node-level network takes these W2V encodings as input, integrating the information within the sub-sequence. Its output, the node-wise averaged attention encodings, referred to as segment encodings, are passed to the segment-level network. We implemented a similar structure for the segment-level network, which integrates segment-level encodings. At the whole-neuron level, we implemented a fully connected layer to output the probability estimation of cell types (Figure 3A; see experimental procedures). Key hyperparameters of the model include the sizes of hidden layer dimensions of W2V and HAN, segment number, and segment length. Settings of hyperparameters are detailed in Figure S2 and Table S1.

We trained and tested the DSM-HAN model on a dataset with 1,282 neuron cells from 12 manually assigned types based on their soma locations and projecting brain structures (Table S3). We used 80% of the data (1,025 cells) for training and the remaining 20% (257 cells) for testing. Through 30 independent training sessions (random sampling of training and testing data), the model achieved 92.76% average testing accuracy. The high class-wise area under the curve (>0.98 in all cases) scores of the receiver operating characteristic suggest high robustness (Figure 3B). We performed ablation studies to test the necessity of using the complex DSM-HAN model. Simpler models including multilayer perceptron and random forest were operated on the flattened W2V encodings (Figure S3), receiving average test accuracy of 36.52% (random forest) and 83.50% (simple multilayer perceptron). The results indicate DSM-HAN as a superior module for classifying neuron sequences.

The information of the input neuron sequences was encoded from two features: the residing brain regions of nodes and their sequential orders. We performed tests to examine whether DSM-HAN was able to learn sequential information that was independent from brain regions. At the segment level, we performed hierarchical clustering for the segment encodings and identified the top 2 clusters for each cell (Figure S1). Segments with close sequential orders were clustered together regardless of the residing brain regions, even for neurons with multiple target regions (e.g., ET_MO neurons). At the neuron level, we reversed sequence orders for each cell and performed clustering together with the original sequences (Figure S4). For most cell types, the reversed and original sequences form distinct clusters, and one sample discriminability tests^23^ (see experimental procedures) indicate clear separation in their native embedding space (Figure S4). The only exceptions were found for IT neurons where the segments’ residing regions are highly invariable. These results indicate that DSM-HAN utilizes the sequential information of projection orders, which further influence the neuron encodings.

We compared the model performance with alternative methods including TFIDF, graph convolutional network (GCN), TextRNN, TextCNN, NBLAST, Sholl analysis, L-Measure, and topological morphology descriptor (Figure 3C). TFIDF is a classic sequential model widely used for feature extraction in document classification.^24^ TFIDF was applied to extract features from neuron sequences, making the dataset a “number of cells” by “number of unique brain regions” matrix (see experimental procedures). We obtained an average testing accuracy of 89.29%. Alternatively, GCN^25^ presents a simple network utilizing a graph structure of data, achieving great classification results on tasks, such as node classification, relation classification, etc. We used the tree structure of neurons as the graph, and the brain region (W2V encodings) as input features, achieving a testing accuracy of 91.02%. TextRNN and TextCNN are both deep models for text classification, and one uses recurrent neural network [RNN] as basic feature extractor, the other uses CNN.^26^^,^^27^ Using the W2V encodings, the testing accuracy is 85.67% for TextRNN and 86.12% for TextCNN. NBLAST calculates the spatial proximity between neuronal segments through structural alignment.^13^ We used NBLAST similarity matrix values as input features for several machine learning algorithms, among which linear-kernel SVM showed the best testing accuracy (72.74%). Sholl analysis characterizes the spatial branching pattern of a neuron by counting the number of its intersections with concentric spheres centered by the soma. We used the intersection profiles as features (see experimental procedures), and trained linear-kernel SVM classification models, showing an average testing accuracy of 66.08%. We used morphological features (see experimental procedures) summarized by L-Measure to characterize neuron morphologies and trained linear-kernel SVM for classification (average testing accuracy, 71.88%). The “persistent homology” analysis couples morphology properties and neuron branching patterns,^14^ which is also known as topological morphology descriptor^15^ (TMD). The radial distance of current node to soma is chosen as the description function to convert neuron structures to persistent diagrams, characterizing neuron morphologies. We obtained average testing accuracy with linear-kernel SVM (68.43%). For each alternative method, we divided training and testing datasets using the same strategy as for DSM-HAN and trained classification models through 30 independent training sessions. Comparison of testing accuracy indicates the significant outperformance of DSM-HAN over alternative methods (Figure 3C).

To investigate the effectiveness of our brain region encodings (from the W2V model), we compared it with direct node coordinates (x-y-z) in the supervised cell-type classification. The two sets of features are tested using both the DSM-HAN and GCN models, and results show the advance of our brain region encodings over node coordinates in both models (Figure S5).

To investigate the generalizability of DSM-HAN for morphological data, we tested its performance for alternative datasets, including a newly released full morphology dataset^28^ with 11 cell types (mostly different from the 12 cell types of the SEU dataset^4^) and local morphologies (dendrites) from the SEU dataset (see experimental procedures). DSM-HAN achieved 92.2% testing accuracy for the 11-cell-type dataset and UMAP of the hidden layer encodings shows distinct separation between cell types (Figure S6). For classification of dendrites, DSM-HAN outperformed alternative methods (Figure S7), although its performance (testing accuracy = 76.4%) was lower than that in the classification tasks of long-projecting neurons. The reduced accuracy can be explained by the highly similar sequence encodings of dendritic types from the same brain regions (e.g., IT_MO and ET_MO). The performance can be further improved by refined brain region assignment (e.g., dividing cortical regions by layers; Figure S7), reaching to 84.7%.

To test the robustness of the DSM-HAN model to the registration deviation, we introduced Gaussian noise to the node coordinates of the testing dataset (257 cells). This perturbation resulted in changes to the brain region assignment of many nodes. The noise level (the standard deviation of Gaussian distribution) was gradually increased from 10 to 1,000 μm, with a 10 μm step size (Figure 3D). Results show that the testing accuracy was stably high (>90%) with a noise level <200 μm. Empirically, the maximum registration deviation for mouse brains is 100 μm.^29^ Thus, the DSM-HAN model is robust to variations introduced by registration and brain region assignment. We further test the robustness of all alternative approaches. Results show that TFIDF and NBLAST were able to maintain their accuracy under spatial noise <100 μm, while the performance of others fell dramatically with increased noise (Figure S8).

### Autoencoder: Concise representation for exploratory studies and data retrieval

Direct cell-cell comparison is important for exploratory studies and data retrieval. As there is no exact node-to-node correspondence between two different neuron reconstructions, it becomes necessary to generate comparable and quantitative representations for them. Here, we adopted a sequence autoencoder as the DSM-AE model. This model aims to learn a latent vector representing input neuron sequence and recover the input sequence from the latent vector (see experimental procedures). The training process optimizes parameters of the model to minimize the difference of recovery and input sequence. Thus, the latent vector representation significantly reduces the data dimensionality while retaining most of the projection information.

**Figure 4:**
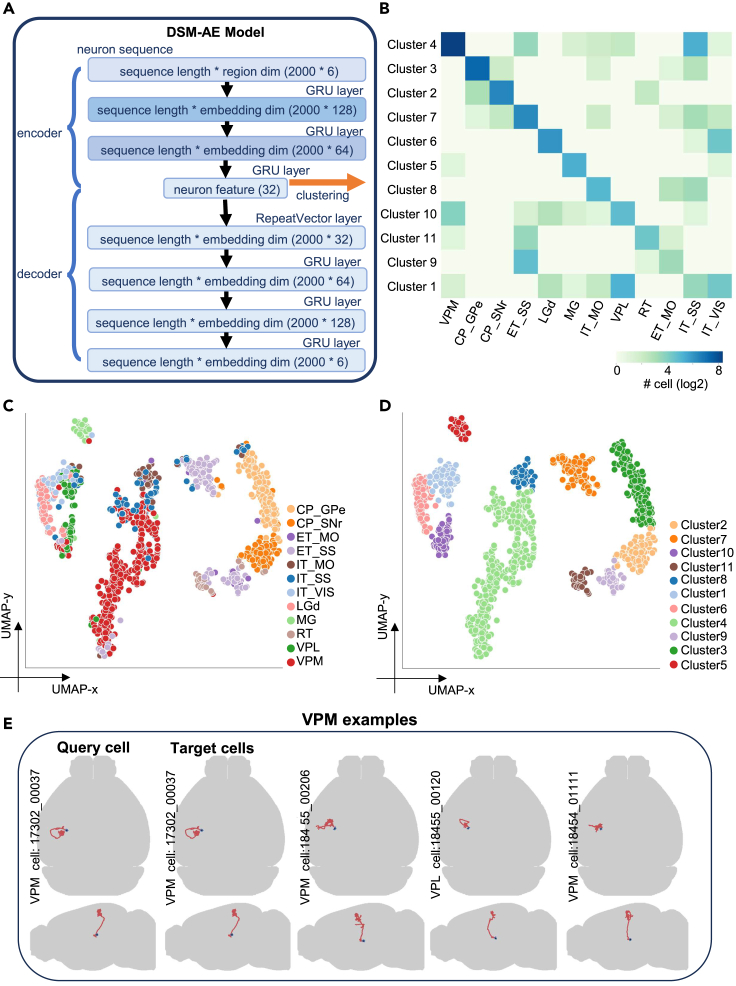
Autoencoder: Concise representation for exploratory studies and data retrieval (A) DSM-AE network structure. (B) Confusion matrix of clusters and cell types. The color bar indicates the 2-based logarithm of cell numbers. (C) Data visualization of the DSM-AE encoding. The neuron sequences are encoded as 32-dimension vectors and projected to 2D space by the UMAP algorithm. Colors indicate cell types (left) or the DBSCAN cluster assignment (right). Only cells above DBSCAN confidence threshold are shown. (D) Morphological data retrieval. The brain-level views of query cells and target cells are reported by the online service.

For applicability evaluation, we applied the model to the 1,282-cell dataset of 12 cell types (see experimental procedures) and generated their DSM-AE representations. DBSCAN^30^ (see Figure S9 for parameter determination policy) was operated on tSNE-transformed DSM-AE representations to identify 12 clusters. We used the adjusted Rand index (ARI) to measure the correspondence between clusters and cell types (ARI = 0.719; Figure 4B). We used the discriminability metric^23^ to measure the separation of 12 cell types in the t-SNE-transformed embedding space (discriminability = 0.888). 2D visualization of the DSM-AE encoding shows that cells from the 12 different classes form distinct sub-populations and the distribution of the clusters match the manually defined projection-based groups (Figures 4C and 4D).

For comparison, we applied the same procedure to evaluate alternative approaches including TFIDF, NBLAST, Sholl analysis, L-Measure, and TMD. We also performed the evaluation for the direct W2V sequence representations (see experimental procedures). To eliminate the possible effect of post-processing methods operated on the native representations, we calculated ARI and discriminability under a variety of post-processing procedures, including standardization, PCA, tSNE, UMAP, ISOMAP, MDS, and LLE (see experimental procedures). For both ARI and discriminability, DSM-AE demonstrated outperformance and higher robustness over alternative approaches, under all post-processing procedures (Table S4).

For exploratory studies in which the number of cell types are unknown and novel cell types may exist, DSM-AE can be combined with DSM-HAN for automated dataset annotation. For identification of novel cell types, we introduced an outlier detection module with 12 outlier detectors. For each known cell type, a detector is a one-class SVM model using DSM-AE encodings as input data (see experimental procedures). After a cell is assigned to a cell type by DSM-HAN, the corresponding detector is applied, and changes its label to “unknown” if the cell is found to be an outlier. As a proof of concept, we applied this approach to the annotation of an external set of full morphology neurons (Janelia dataset; 1,002 neuron cells^3^), 61% cells of which belong to novel cell types (Table S5). We evaluated the outlier detection performance by comparing its predictions with the manually curated labeling. The median of F1 score was 0.54 (VPM = 0.71; VPL = 0.33; IT_MO = 0.85; ET_MO = 0.72; IT_VIS = 0.55; ET_SS = 0.07; IT_SS = 0.38). We examined poorly performing detectors and found that most of the cells were assigned to cell types with similar morphology (e.g., VPM assigned as VPL, ET_MO assigned as ET_SS). We summarized the training and testing results in Table S6.

The DSM-AE representations are further reduced to 2D using a UMAP algorithm, resulting in a 2D reference atlas. User-uploaded morphological data (query data) are projected to the 2D reference atlas for determining cell types according to the similarity with reference data points. In addition, the most similar single cells (target data) and their horizontal/vertical views are reported for visual inspection (Figure 4D). Also, more examples including the least similar cells from non-target neuron classes are also provided, demonstrating the diverse morphological types in the dataset (Figure S10). The website (see data and code availability) also provides a series of tools including interactive visualizations of W2V brain region encodings, 2D reference data atlas, and 3D single-cell morphologies.

### Subtyping of neurons cross-validated by analysis of projection patterns

**Figure 5:**
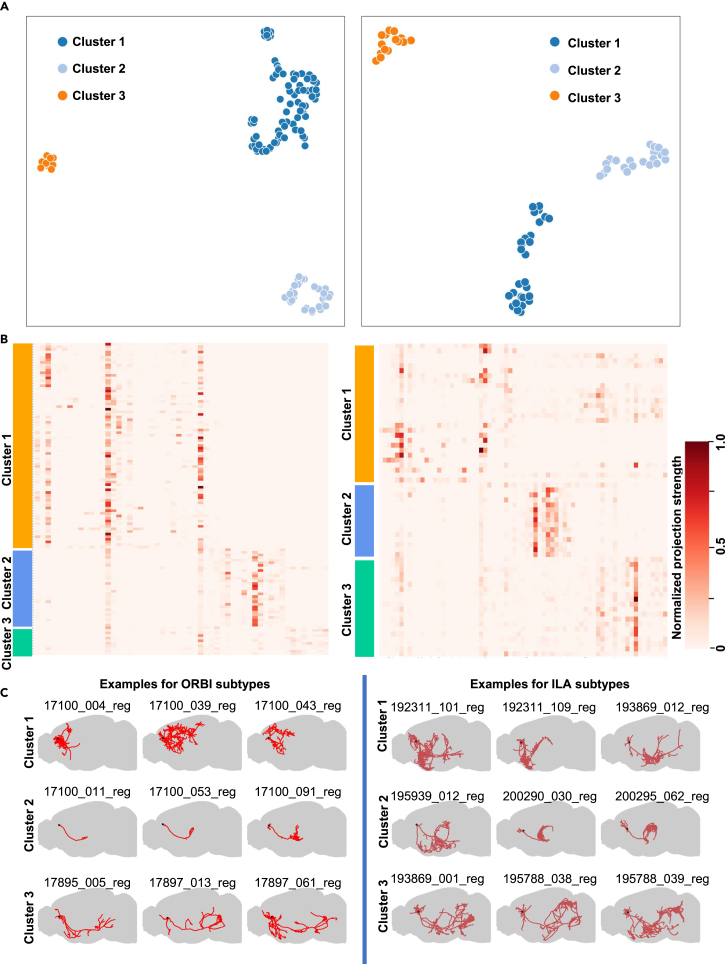
Sub-typing of neurons cross-validated by analysis of projection patterns (A) Cell sub-typing derived from DSM-AE embedding (left, ORBl cells; right, ILA cells). (B) Projection patterns of DSM-AE sub-types. Rows, cells grouped by DSM-AE clustering; columns, target brain regions. The projection strength was defined as the axon length in target brain regions normalized between 0 and 1. (C) Visualization of neuronal axon reconstructions of three examples for each DSM-AE sub-type.

It is intriguing to explore previously unknown sub-types of cells. To examine whether the DSM-AE model may facilitate the discovery of neuronal sub-types, we cross-validated it with third-party neuron morphology datasets. Particularly, we focused on two major brain structures: the orbital area, lateral part ORBl, and the infralimbic area (ILA). For each of them, we trained a DSM-AE model that characterized cells with 32-dimension embeddings. These embeddings formed distinct sub-populations (Figure 5A), suggesting different previously unknown sub-types in the respective cell class. We cross-validated these sub-types of cells by examining their projection strength patterns manifested by the respective axon length calculated from neuron reconstructions in a recent independent study^28^ in target brain regions. We found clear clustering patterns (Figure 5B). Our results show high reliability of the DSM-AE sub-types (ORBl: ARI = 0.989; ILA: ARI = 0.381; Table S7). Visual inspection of the 3D morphology of example neurons also confirmed these sub-types (Figure 5C).

We applied the same procedure to explore morphological variations in all the 12 cell types from the SEU dataset. The region-wise projection strength drove the clustering results for most cell types (e.g., IT_SS cells clustered by projections in sub-regions such as SSp-bfd and SSp-m; Figure S11). Unlike the ORBI and ILA types where distinct sub-clusters were identified, most of the 12 cell types were less heterogeneous and showed continuous distributions in the embedding space. In addition to variations in projection strength, DSM-AE was able to capture essential characteristics of cell identity. For example, re-clustering of IT_SSp-bfd neurons demonstrates a morphological continuum where L2/3, L4, and L5 cells are distributed in different portion. A similar pattern was observed for the IT_SSp-m neurons (Figure S11).

### Analysis of co-expression genes in connected brain regions

**Figure 6:**
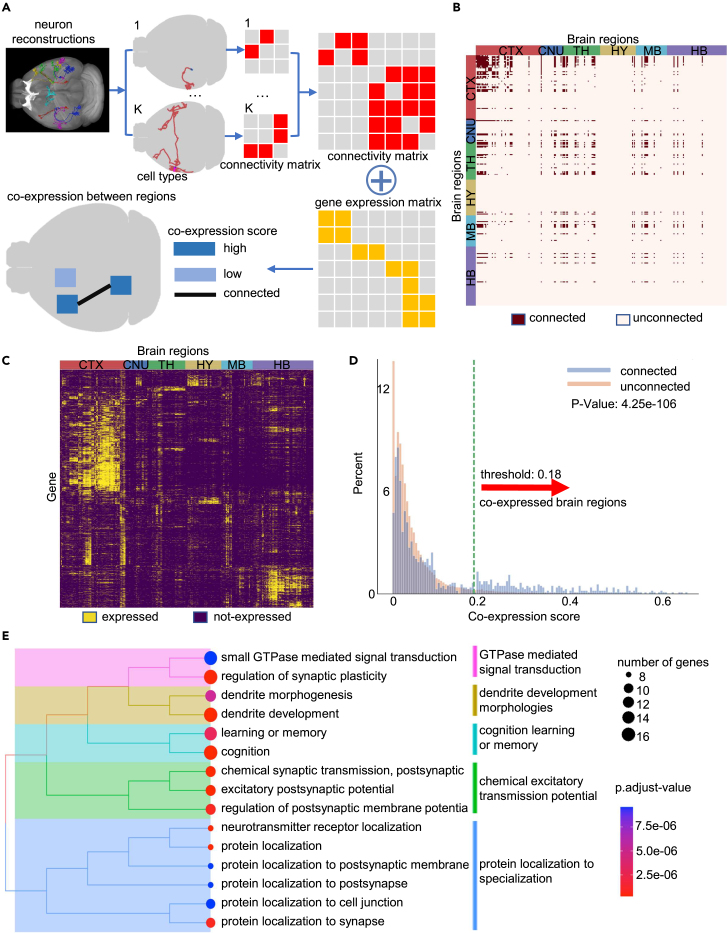
Analysis of co-expression genes in connected brain regions (A) Illustration of a pipeline of co-expression analysis with the following steps: (1) input data as the morphology of 1,282 neurons, (2) identification of cell types using our DMS method, (3) determination of connected brain regions at cell-type levels, (4) combination of connectivity matrix and gene expression matrix at the whole-brain level, and (5) comparison of co-expression scores between connected and unconnected regions. (B) The brain-region-wise connectivity matrix. Rows and columns indicate brain regions grouped by their major brain regions, including CNU (cerebral nuclei), CTX (cerebral cortex), TH (thalamus), MB (midbrain), HY (hypothalamus), and HB (hindbrain). (C) The binary gene-by-brain region expression matrix. Colors indicate identified gene expression state (see experimental procedures). (D) Histogram for co-expression scores of connected and unconnected brain regions (Kolmogorov-Smirnov two-sided test). Red arrow: the 95th percentile of unconnected regions. (E) Functional enrichment analysis of co-expression genes between the 2,251 connected region pairs with high co-expression scores. GO tree plot results show the clustering of the top 15 enriched functional terms (adjusted p values, hypergeometric test, “BH” correction).

Spatial gene expression analysis has provided insights to resolve the organization of functional neural circuits.^31^^,^^32^ The complexity of 3D gene expression distribution was previously studied from the perspective of cell types and brain regions.^31^ As the gene expression of any brain area is affected by local cell bodies as well as the neurites from distal projecting neurons, it is of great importance to examine the contribution of brain-region connectivity to the spatial gene expression pattern. To our knowledge, such analysis has not been reported, likely being limited by the lack of clear definition of brain connectivity. Here, we propose to identify inter-connected brain regions using cell types defined by our DMS method. We first applied DMS-AE and DBSCAN to the 1,282-neuron dataset, identifying 11 clusters (see experimental procedures). For each cell type, we defined the projecting source and target regions as “inter-connected” (Figure 6A; see experimental procedures). Then we integrated the cell-type-level connections to generate a joint connectivity matrix, which can be combined with spatial gene expression data for further analysis (Figure 6B; see Table S8 for the connectivity matrix).

With the connectivity matrix, we asked whether inter-connected brain regions tend to show similar expression patterns. We identified expressed genes across these brain regions using the Allen Brain Atlas^21^ and obtained a region-wise gene expression matrix that showed co-expression modules among neighboring regions from the same major brain regions (Figure 6C; see experimental procedures). We defined the co-expression score as the Jaccard similarity coefficient between the expressed gene sets of any two regions (see Table S9 for the co-expression matrix). Connected region pairs had substantial co-expression scores (p = 4.25e−10^6^, two-sided KS test; Figure 6D). Similar co-expression patterns were observed even when we excluded region pairs from the same major brain region (p = 2.92e−24, two-sided KS test; Figure S12A) to avoid the effect of co-expression between neighboring regions. We selected 2,251 connected region pairs whose co-expression scores were above the 95th percentile of unconnected ones (Figure S12B and Table S10). We found that the co-expression genes of the 2,251 pairs were highly enriched in top-row functional terms including synaptic specification, dendrite development, and cognition (Figure 6E). Synaptic transmission has been reported as an over-represented gene set that supports both structural and functional inter-regional connectivity.^33^^,^^34^ Our analysis identified brain-specific angiogenesis inhibitor 1-associated protein 2 (Baiap2) as the most frequently co-expressing gene between connected regions. BAIAP2L2 (a paralog of BAIAP2), which induces planar or curvature membranes, was previously reported to be reflective of structural connectivity of human brains.^35^ Among the top 200 regional co-expressing genes, we also identified genes related to ion channels (Camk2a, Camkk1, Kcnj4), neuronal process formation (Enc1), and neurotransmitters (Syt16). Remarkably, a general survey of expressed genes in the brain atlas suggested more broad functional terms including organelle localization, histone modification, and signal transduction (Figure S12C). Compared with these broader “background” functions that might be contributed by “housekeeping” genes or non-neuronal cell types in the brain,^31^ our results in Figure 6E suggest previously unreported patterns that cell-type-specific brain connectivity is influential to the spatial gene expression, likely through the co-expression of genes that support neuron-specific functions.

## Discussion

Complete morphology of long-range projecting neurons is crucial for deciphering the diversity of cell types and for understanding organizational principles of brain connectivity. The unique and complex nature of morphological structure implies difficulties for data analysis. In this work, we introduce a sequential model for feature extraction from full neuronal morphology, which enables efficient cell-type classification, morphological clustering, and data retrieval. We provide a series of computational tools that outperform existing ones in accuracy and robustness. These tools are available as online services for researchers with or without computational backgrounds.

Compared with the transcriptome data based on gene sequencing, it is challenging to define morphological features that effectively represent cell-type-related characteristics. Although some feature sets (e.g., L-Measure^9^) are more influential, there is limited consensus on a reasonable practice for feature definition. In previous studies, we defined cell types by where their projection initiates and terminates.^4^ Such projections form directional trajectories in the brain, which turned out to be highly consistent within a cell type. The data structure of such trajectories shares a lot of similarities with texts, the basic structure of which is also arrays of nodes. For a paragraph of text, a node is a word. For the morphology of a neuron, a node is a point along a branch. Such arrays are logically (for texts) or physically (for neurons) connected, forming branching structures. In the field of natural language processing, a series of classical techniques enable tasks including texts understanding and generation. The similarity of data structure renders NLP techniques applicable for the study of neuronal projection paths.

Characterization of single-neuron morphologies provides valuable insights for the understanding of neural circuits. Like single-cell transcriptomes, analysis of neuronal morphologies at the level of clusters formed by similar cells is crucial for information mining. In this study, we demonstrate the application of our DSM model in finding new sub-types and the results were consistent with double-blind classification by experts. The identified sub-types may serve as anchors for crossmodality comparisons to achieve comprehensive definition of cell types. Accurate morphological classification also provides an appropriate granularity for crossmodality joint analysis. In this study, we used DSM-identified morphological clusters to define brain region connections, which avoided the lack of resolution at population level and susceptibility to false positives at the single-cell level. The inter-connected regions show significantly higher levels of gene co-expression, which is valuable to explain spatial gene expression data from the perspective of neural connections. More biological insights might be revealed by combination with spatial gene expression data at the single-cell level.

Although our sequential representation and deep learning models showed high performance in the morphological classification tasks, several limitations and future improvements are worth noting. First, the method is specifically designed for long-range projecting neurons. It is not applicable for the classification of neurons without long-range projections (e.g., most interneurons). Second, our model neglects some local morphological features (e.g., segment curvature, branching angle, etc.), which also potentially bear information of cell identity.

There are several future directions related to our model. First, motif analysis is a classical approach in the study of DNA sequences. It is interesting to identify repetitive and characteristic sub-structures of a cell type that might be related to both neuronal identity and development history. Second, the DSM enables the possibility of cross-species comparison. A metaphor for this is comparing articles written in multiple languages. The W2V model enables identification of paralogs of brain regions and the projection path analysis enables comparison of cell-type paralogs. This will be made possible with the availability of full-morphology data from other species, the most possible one being the monkey in the future.

## Experimental procedures

### Resource availability

#### Lead contact

The lead contact for this work is Hanchuan Peng (h@braintell.org).

#### Materials availability

This study did not generate new unique materials.

#### Data and code availability

The codes, preprocessed data files, and supplemental files are archived on the Zenodo repository (https://doi.org/10.5281/zenodo.8186904 https://zenodo.org/record/8188431).^36^ We also provide a python package integrating DSM models, which can be easily installed by a “pip install DSM-tools” command; source codes are also provided on Zenodo. To visualize neuron morphology and retrieve neurons based on morphological similarity, we provide an open-source online service.^36^

### Transform neuron topological structure to sequence

Tree-like structures can be transformed into sequences through tree traversal algorithms. Here, to make the local morphologies a continuous sub-sequence and unfold these local morphologies along the neuron projection (from soma to distal arbor), we apply a depth-first traversal algorithm with designed traversal order.

While the soma node is the only node that could have more than two child nodes, to avoid multifurcations the soma node is discarded from the neuronal structure in advance, and its removal converts the neuron structure into several binary trees. Afterward, we apply depth-first traversal strategy on each tree to generate the corresponding sequences and concatenate them into one sequence representing the full neuron structure. This depth-first search strategy keeps adjacent connected nodes in the tree structure as close as possible in the sequence representation. The rules for traversal were set as follows: sub-trees with fewer leaf nodes and shorter path are visited first. Upon iterative traversal through the tree nodes, we append each visited node to the sequence. Finally, instead of using a series of spatial coordinates directly, we encode each node with the ID of the brain region where it is located in the Common Coordinate Framework v.3 (CCFv3) 3D reference space^20^ (resolution, 25 μm).

### Preparation and pre-processing of dataset

The major classification and clustering tasks are conducted on a dataset of 1,282 neuron cells with labels, including 12 classes determined by their projection path in the CCF brain. Original reconstructed neurons have been published in our previous work (SEU dataset),^4^ and preprocessed datasets were stored in the Zenodo repository.^36^ The classes consist of CP_SNr (100 cells), CP_GPe (180 cells), VPM (378 cells), ET_SS (159 cells), IT_SS (97 cells), VPL (80 cells), LGd (78 cells), MG (50 cells), IT_VIS (48 cells), IT_MO (48 cells), RT (33 cells), and ET_MO (31 cells).

The local morphologies (dendrites) dataset also come from the separation of local dendrites from the full morphologies in the same 1,282 neuron cells including the same 12 classes.

For the newly released dataset with more than 6,000 neurons,^28^ we categorized them into 11 cell types according to their soma regions, and sampled 100 cells from each cell type, forming a dataset with 1,100 cells (Table S11).

For training brain regions, we used 1,074 cells (from the 1,282 cells) as a training dataset and augmented it by adding four types of spatial Gaussian noise (mean = 0 μm, standard deviation = 5, 10, 15, and 20 μm, respectively). For each node, a random displacement is added to its spatial coordinate. After the augmentation, the total dataset contained 5,370 neurons.

To standardize the neuron reconstructions, we performed two preprocessing steps: small neuron segments, the length of which is less than 10 μm, were removed and the distance between adjacent connected nodes was readjusted to 20 μm (using “resample swc”, a Vaa3D built-in plugin,^37^ v.3.601).

### Train brain regions to dense vectors

To utilize brain regions in neuron cell type analysis effectively, a proper method is needed to vectorize regions. One-hot encoding encodes categorical features by creating a binary column for each category. However, one-hot encoding not only fails to indicate the relationship of encoded vectors but also triggers dimension explosion if there are too many categories in a dataset. Thus, in natural language process tasks, W2V is commonly used to train word embeddings from one-hot encoding to dense vectors in a corpus.^22^ The intuitive idea behind W2V is to gather similar words and disperse irrelevant words in their latent vector space. Since one-hot encodings of words are similar to brain regions in our case, it is easy to extend this method to train brain region encodings.

There are multiple model architectures to train W2V encodings. As illustrated in Figure 2A, we selected continuous bag-of-words as the W2V architecture. Initially, each word is encoded as 1-of-V vector using one-hot encoding, where V is the size of the vocabulary (all words in the corpus). Instead of feeding a whole sequence as input, we see each word (center word) and its neighboring words (context words, size = N) as an input-output pair, transferring a complete sequence into sub-sequences. The input layer projects context words (N × V data matrix) to the linear hidden layer (D-dimension latent space), using a weight matrix W (V × D, shared by N words). At the hidden layer, the N words embeddings are averaged as the center word representation (D-dimension, dense vector), which is also used to predict the 1-of-V vector of the center word. Given a one-hot encoding of input word, the W2V model gives its dense vector by multiplying matrix W.

For the classification task of local morphology (dendrites) from the 1,282 SEU dataset, we further divided cortical regions to laminar levels, finding that the detailed separation of brain regions can improve the performance of local morphology classification by 8% (Figure S7).

The W2V model is available in an open-source python library, Gensim^38^ (v.3.7.0; API: gensim.models.Word2Vec; parameters; Table S1) for the training of vector embedding.

### DSM-HAN for morphology classification

We explored alternative models to reach higher and more robust performance in classification. HAN^19^ is a supervised classification algorithm to classify documents in NLP. Unlike traditional document classification treating the whole document as one continuous word sequence, HAN focuses on the structure of the document and builds representations of sentences that are then aggregated into a document representation.

The intuition of HAN is not classifying documents based on isolated words, but rather relating the task with the interaction of words. By transplanting the idea to neuron sequence, we introduce the DSM-HAN model, which decomposes a neuron sequence (a document) into segments (sentences) according to the topology of neuron structure, and treats each segment as a sub-sequence, representing local morphologies. The number of segments in each neuron sequence and the segment length (the number of nodes in each segment) are determined by their distributions (Figure S2).

The model architecture is summarized in Figure 3A. It has three parts: a word-level network (blue boxes), a sentence-level network (yellow boxes), and a classification network (orange boxes). The word- and sentence-level networks have basically the same architecture including a word sequence encoder (GRU, gated recurrent unit^39^), a word attention layer, and an average layer. The classification layer consists of several fully connected layers, outputting the probability estimation of cell types.

We used GRU to embed sequence data, and the attention layer to evaluate the importance of each state in a sequence and to add weight to them. The GRU belongs to the RNN family, with the ability to encode information from sequence data. Compared with traditional RNN, the GRU has two types of gates, reset gate $r_{j}$ and update gate $z_{j}$, which are used for solving gradient vanishing in RNN-related training. At the *j*-th state of a sequence, the current hidden state $h_{j}$ is computed by

$$h_{j}=z_{j}h_{j-1}+\left(1-z_{j}\right)\tilde{h_{j}}$$

where update gate $z_{j}$ decides the proportion of current candidate hidden state $\tilde{h_{j}}$ to previous hidden state $h_{j-1}$ in current hidden state. The $\tilde{h_{j}}$ is computed as:

$$\tilde{h_{j}}=tanh\;\left(W_{h}x_{j}+U_{h}\left(h_{j-1}⊙r_{j}\right)+b_{h}\right)$$

where reset gate $r_{j}$ balances the contribution between current input information and previous state information to candidate hidden state $h_{j}$.

The gates $r_{j}$ and $z_{j}$ are computed by

$$r_{j}=\mathrm{sigmoid}\left(W_{r}x_{j}+U_{r}h_{j-1}+b_{r}\right)$$

$$z_{j}=\mathrm{sigmoid}\left(W_{z}x_{j}+U_{z}h_{j-1}+b_{z}\right)$$

The attention layer, origin from the attention mechanism, gives prominence to important states in a sequence by assigning higher weights to them. The mechanism filters out fewer relative words and causes contributing words to dominate in tasks. For the current state $h_{j}$, we have

$$u_{j}=tanh\;\left(W_{w}h_{j}+b_{w}\right)$$

$$\alpha_{j}=\frac{\exp\;\left(u_{j}^{T}u_{w}\right)}{\sum_{j}\exp\;\left(u_{j}^{T}u_{w}\right)}$$

$$s=\sum_{j}\exp\;\left(\alpha_{j}h_{j}\right)$$

where $u_{j}$ is the embedding of each state h by one-layer multilayer perception, denoting the importance of each state, and $\alpha_{j}$ is the normalized weight of the state $h_{j}$. *s* is the weighted sequence embedding as the final output, and subscripts *W*, *U*, and *b* refer to trainable weights.

We built the DSM-HAN model using a TensorFlow^40^ (v.2.4.0) framework.

### DSM-AE for morphology representation

Traditionally, neuron morphologies are encoded by selecting features that are highly expert dependent and suffer from information loss inevitably. Here, we introduce the autoencoder model, which is an unsupervised neural network built for representation learning. The model aims to learn a latent representation of input neurons and reconstruct the original morphology from the latent representation.

The autoencoder architecture is summarized in Figure 4A. The model has two parts: encoder and decoder. The encoder part takes a neuron sequence as input, and encodes it into a reduced dimension vector, representing the neuron morphology; and the decoder is responsible for reconstructing the original input sequence from the reduced vector. Both encoder and decoder are composed of GRU layers, and they are connected by a RepeatVector (RV) layer. The RV layer simply repeats its input data, and we used it here to recover the sequence length. The implementation of DSM-AE is realized by TensorFlow^40^ (v.2.4.0) framework.

We clustered the neurons with the encoder outputs. The clustering is performed using the Density-Based Spatial Clustering of Applications with Noise (DBSCAN) algorithm^30^ under strict clustering policy (Figure S9). Specifically, the noise recognized by DBSCAN was removed first. This part is also realized by the “scikit-learn” python package^41^ (v.1.0.2; API: sklearn.cluster.DBSCAN and sklearn.metrics.adjusted_rand_score; parameters, see clustering policy: Figure S9).

Quantification of the separation of 12 cell types is evaluated by the discriminability.^23^ This part is realized by the python package “hyppo”^42^ (v.0.3.2; API: hyppo.discrim.DiscrimOneSample; parameters, default).

The post-processing methods include z-score normalization, PCA, tSNE, UMAP, ISOMAP, MDS, and LLE, which are implemented by two python packages, including scikit-learn^41^ (v.1.0.2; API: sklearn.preprocessing.StandardScaler, sklearn.decomposition.PCA, sklearn.manifold.Isomap, sklearn.manifold.LocallyLinearEmbedding, sklearn.manifold.MDS, and sklearn.manifold.TSNE; parameters, default) and the “umap-learn” python package^43^ (v.0.5.3; API: umap.UMAP; parameters, default).

To provide predictions of cells similar to the input of the model, we converted the input cell sequence into a 32-dimension vector using the autoencoder model and used the Euclidean distance to recommend the top 4 cells.

### Hyperparameter determination

To achieve high performance of DSM models, we tested rounds of hyperparameters and summarized the key results (Figure S2; Table S1).

For the hidden layer dimension of the W2V model (testing range, 1–80), we found that the validation accuracy of DSM-HAN exceeded 90% with hidden layer dimension >3 (Figure S2).

For DSM-HAN learning rate (LR), we fixed other parameters and trained DSM-HAN under different LR values (0.1, 0.01, 0.001, and 0.0001). With LR = 0.1, the training process resulted in overshooting where the training loss dramatically increased. With LR = 0.0001, the rate of convergence was low as the validation loss was still decreasing after 300 training iterations. With LR = 0.01 or 0.001, convergence was observed between 50 and 100 iterations. We conclude that LR is a crucial parameter for achieving high classification accuracy (Figure S13). The training of DSM-AE also follows the same procedure and shows similar results (Figure S13).

The hierarchical attention network is a commonly used model in the NLP area. The choice of the number of layers and dimensions has been in a previous study,^19^ and we downsize the two parameters considering the capability of our device (GTX 2060Ti; Figure S2). We also suggest that users explore new combinations of these tunable parameters with the major design unchanged (Table S1).

We further tested a series of hidden layer dimensions for the DSM-HAN classification network, finding that the hidden layer dimensions hardly affect the final training results (Figure S13).

### TFIDF for morphology classification

In the field of natural language processing, the basic form of data is the sequence. By transforming neuron reconstruction data to sequences, we can extend numerous methods from the NLP area to a cell-type classification task.

The acronym TFIDF stands for term frequency and inverse document frequency for a word. Term frequency is the number of times that a term occurs in a document (a neuron sequence), indicating the importance of the term to the document, and inverse document frequency is defined to measure how unique the word is to the document in documents, calculated by dividing the total number of documents by the number of documents containing the word and then taking the logarithm of that quotient.

To perform feature extraction, neuron sequences are regarded as documents, which were converted to a matrix of TFIDF features (number of documents × number of words), and then machine learning algorithms such as SVM can be used on the TFIDF matrix. The feature extraction was implemented using scikit-learn^41^ (v.1.0.2; API: sklearn.feature_extraction.text.TfidfVectorizer; parameters, default).

### Comparison with alternative approaches

The TMD framework^14^^,^^15^ provides a flexible framework to vectorize neuron morphologies. In this study, we used the TMD python package (v.2.2.0; API: tmd.methods.get_persistence_diagram; parameters, default), using “radial distance” as description function, to calculate the TMD features (see https://github.com/BlueBrain/TMD for details).

NBLAST^13^ provides a direct cell-cell comparison method by measuring pairwise neuronal similarity. We used the “NBLAST” R package (v.1.6.5; API: nblast; parameters, v.2, other parameters, default). We obtained the similarity between 1,282 cells, generating a similarity matrix (1,282 × 1,282).

The L-Measure features were acquired by “global_neuron_feature”, a plugin of Vaa3D^37^ (v.3.601). We collected 11 features from the results (Table S12).

The Sholl analysis^44^ were performed by a python script “sholl analysis.py”, which is archived on the Zenodo repository^36^ (https://doi.org/10.5281/zenodo.8186904, see “neuron2seq_for_developer” folder; parameters, r_max: 10,000, and steps: 10).

Linear-kernel SVM, for neuronal morphology classification, is realized by the scikit-learn python package^41^ (v.1.0.2; API: sklearn.linear_model.SGDClassifier; parameters, loss hinge; others, default).

TextCNN^27^ was realized with three convolution layers (steps 2, 3, and 5), and followed by a “concatenate” layer. We used the concatenated vectors for cell classification. The codes are developed by TensorFlow^40^ (v.2.4.0).

TextRNN^26^ was realized with two GRU layers (dims 128 and 64) and followed by two densely connected layers for classification. The codes are developed by TensorFlow^40^ (v.2.4.0).

GCN^25^ was realized by three simple GCN layers (dims 128, 256, and 128), followed by a “global_mean_pool” layer. We used the pooling embeddings as neuron embeddings for classification. The codes are developed by Pytorch-Geometric^45^ (v.2.1.0).

### Outlier detection and automated dataset annotation

To identify unknown cell types, we developed an outlier detection module, combining both the DSM-AE and DSM-HAN models. Our results using DSM-HAN showed that we can provide a reliable cell-type identification for input cells within the 12 projections we studied, but classifying cells beyond the 12 classes was an unsolved problem. To address it, we trained 12 extra one-class SVMs as outlier detectors to correct the DSM-HAN predictions, assigning them to “unknown types” accordingly.

Taking the DSM-AE embeddings as the training dataset, we trained these one-class SVMs, predicting whether each cell is inside-class or outside-class for each cell type. We implemented the one-class SVMs using scikit-learn (v.1.0.2; parameters, default).

### Analysis of co-expression genes in connected brain structures

We used a trained DSM-AE model to obtain embeddings of 1,282 cells, and clustered them using the DBSCAN algorithm (see Figure S9 for clustering policy; see Table S13 for unsupervised labeling). For each cell type, we sorted their target brain regions by projection strength, which is averaged over each target brain region; and, by accumulating the first N averaged projection strength until 90% of the total, we defined the first N brain regions as interconnected within this cell type (Figure S14). To binarize gene expression in target brain structures, for each Allen Brain Atlas^21^ experiment we determined the threshold of gene expression as mean + 1 × standard deviation. To perform the functional enrichment analysis, we used the top 200 most frequently co-expressed genes or 6,169 expressed genes (Table S14) as input gene set. We used the enrichGO function of R package clusterProfiler (v.4.4.1).

## Supplemental information

Document S1. Figures S1–S14

Table S1. Parameters and detailed description for DSM models To help reproduce our results, we provided the training details for three deep models, the hierarchical attention network, the word2vec model, and the autoencoder model.

Table S2. Brain region vectors encoded by word2vec model Each brain region is assigned with a six-dimension vector, and these brain region features can be reused in other studies.

Table S3. Dataset details Our training datasets come from SEU neuron reconstruction. According to the dataset composition used in the hierarchical attention network, the word2vec model, and the autoencoder model, each cell is marked as Y or N.

Table S4. Measurements of ARI and discriminability on DSM-AE representations The correspondence of clusters and cell types is evaluated by ARI, and we calculate the ARI metric under alternative post-processing methods. The quantification of DSM-AE representation is evaluated by discriminability, which also takes the post-processing method into consideration.

Table S5. Summary of the datasets and model predictions on the SEU swc dataset (n = 1,282) and the Janelia swc dataset (n = 1,002)

Table S6. Outlier detector performance An outlier detector is trained to filter unknown cells (not belonging to the 12 classes). The training is performed on the SEU dataset, and the testing is on the Janelia dataset.

Table S7. Dataset detail of unreported sub-types, including two sheets for ORBl and ILA Each cell is assigned with a DSM-AE clustering label and a cross-validated label.

Table S8. The connectivity matrix for whole mouse brain regions Rows and columns indicate brain regions, and values are binary, where “0” represents that two brain regions are unconnected and “1” represents connected.

Table S9. The co-expression score matrix for whole mouse brain regions Rows and columns indicate brain regions, and values are co-expression scores.

Table S10. 2,251 connected region pairs with strong co-expression scores, their major brain regions, and the number of co-expression genes

Table S11. Dataset detail of 1,100 cells from the newly released dataset

Table S12. Summary of 22 L-Measure features used in this study

Table S13. Unsupervised labeling of DSM-AE and DBSCAN for 1,282 cells Each cell is assigned to a cluster, and we built the inner-connection of brain regions based on these clusters.

Table S14. 6,169 genes with over 40 expressed brain regions from 2,251 connected region pairs By counting the co-expression regions for each gene, we selected the top 200 most frequently co-expressed genes as an input gene set for functional enrichment analysis.

Document S2. Article plus supplemental information
