# Supplementary material for: DSM: Deep sequential model for complete neuronal morphology representation and feature extraction
Source: Patterns (N Y). 2023 Dec 13;5(1):100896. doi: 10.1016/j.patter.2023.100896 (PMC10801254; doi:10.1016/j.patter.2023.100896)
Supplement: Document S1. Figures S1–S14 [file mmc1.pdf]

Patterns, Volume *5*

Supplemental information

DSM: Deep sequential model for complete neuronal morphology representation and feature extraction

Feng Xiong, Peng Xie, Zuohan Zhao, Yiwei Li, Sujun Zhao, Linus Manubens-Gil, Lijuan Liu, and Hanchuan Peng

Supplemental Information

Supplemental Items

Supplemental Figures

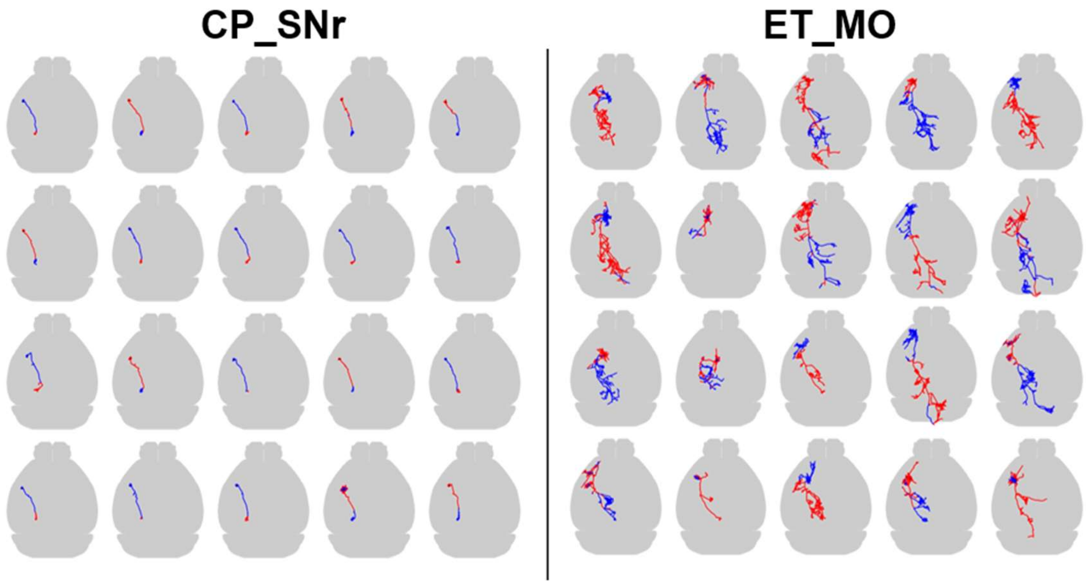

Figure S1. HAN segment-level encodings

Hierarchical clustering was performed for the HAN segment-level encodings for each cell, resulting in two clusters of segments corresponding to the first division of the hierarchical tree. Here we display the top-views of 40 neurons from two cell types, CP_SNr and ET_MO, and segments from same cluster are decorated by same color.

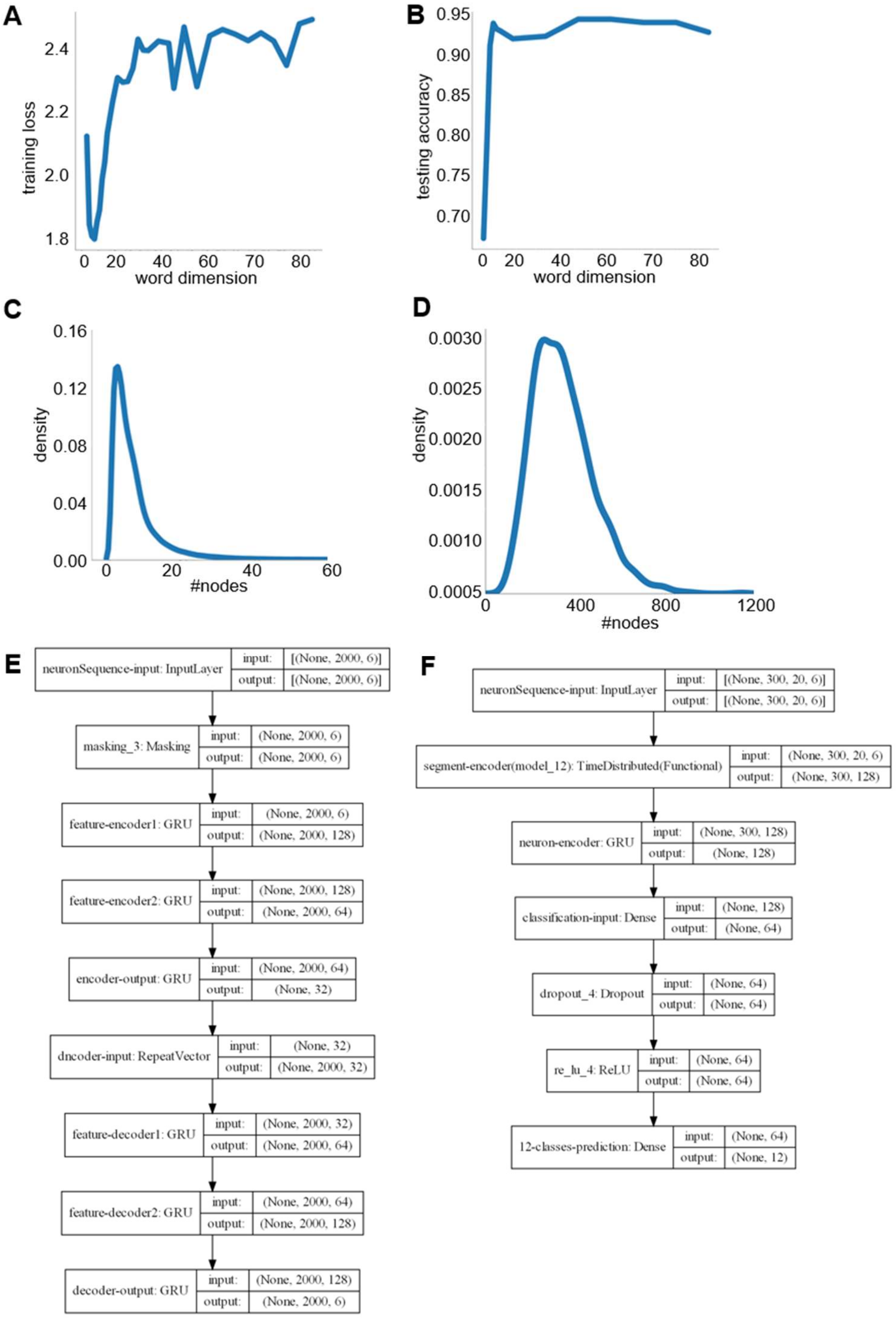

Figure S2. Training details of word2vec

A. Training loss of word2vec. X-axis indicates the dimension of hidden layer (the size of w2v encodings); Y-axis indicates training loss in the latest epoch. B. Testing accuracy of DSM- HAN under different W2V dimension. X-axis indicates the dimension of hidden layer (the size of w2v encodings); Y-axis indicates the testing of accuracy for DSM-HAN classification. C. The distribution of segment length. We used the mean value as the aligned segment length. D. The distribution of the number of segments in each neuron. We used the mean value as the aligned segment number. E. Architecture of DSM-HAN in this study. F. Architecture of DSM-AE in this study.

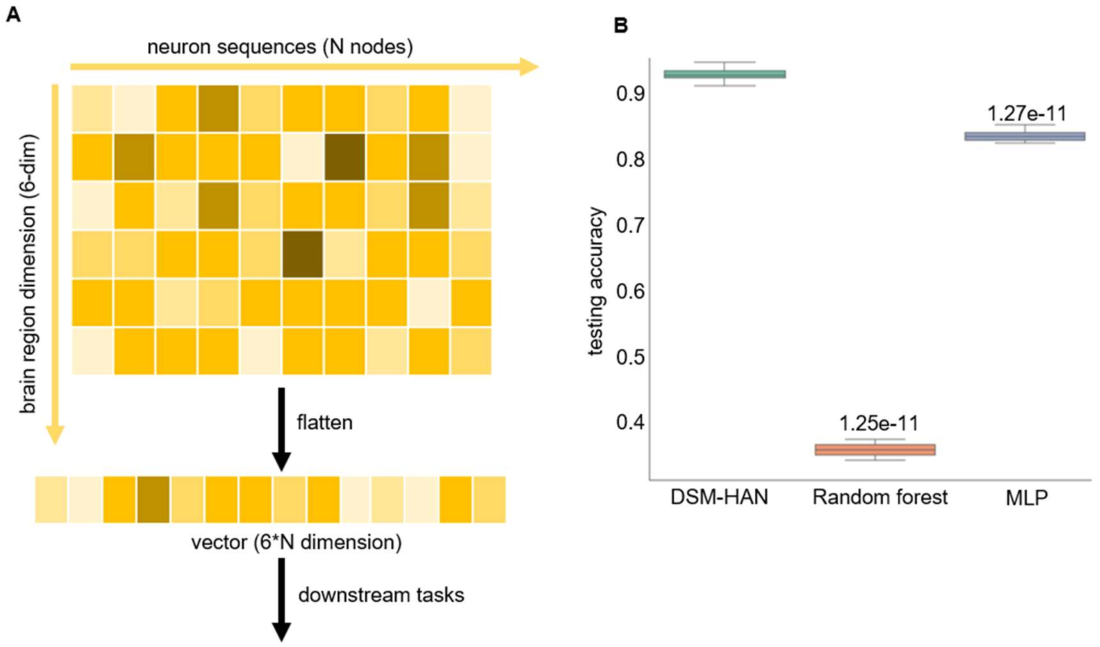

Figure S3. Comparison between HAN and simple classifiers on direct WV2 sequences

A. Flatting word2vec encoding sequences as vectors, we applied downstream analysis, including classification, clustering, and representation analysis on them. B. The classification comparison between DSM-HAN and simple classifiers, including random forest, and multilayer perceptron (numbers above box: P-Values of the Mann-Whitney U rank test (one-side) on test accuracy between DSM-HAN and others).

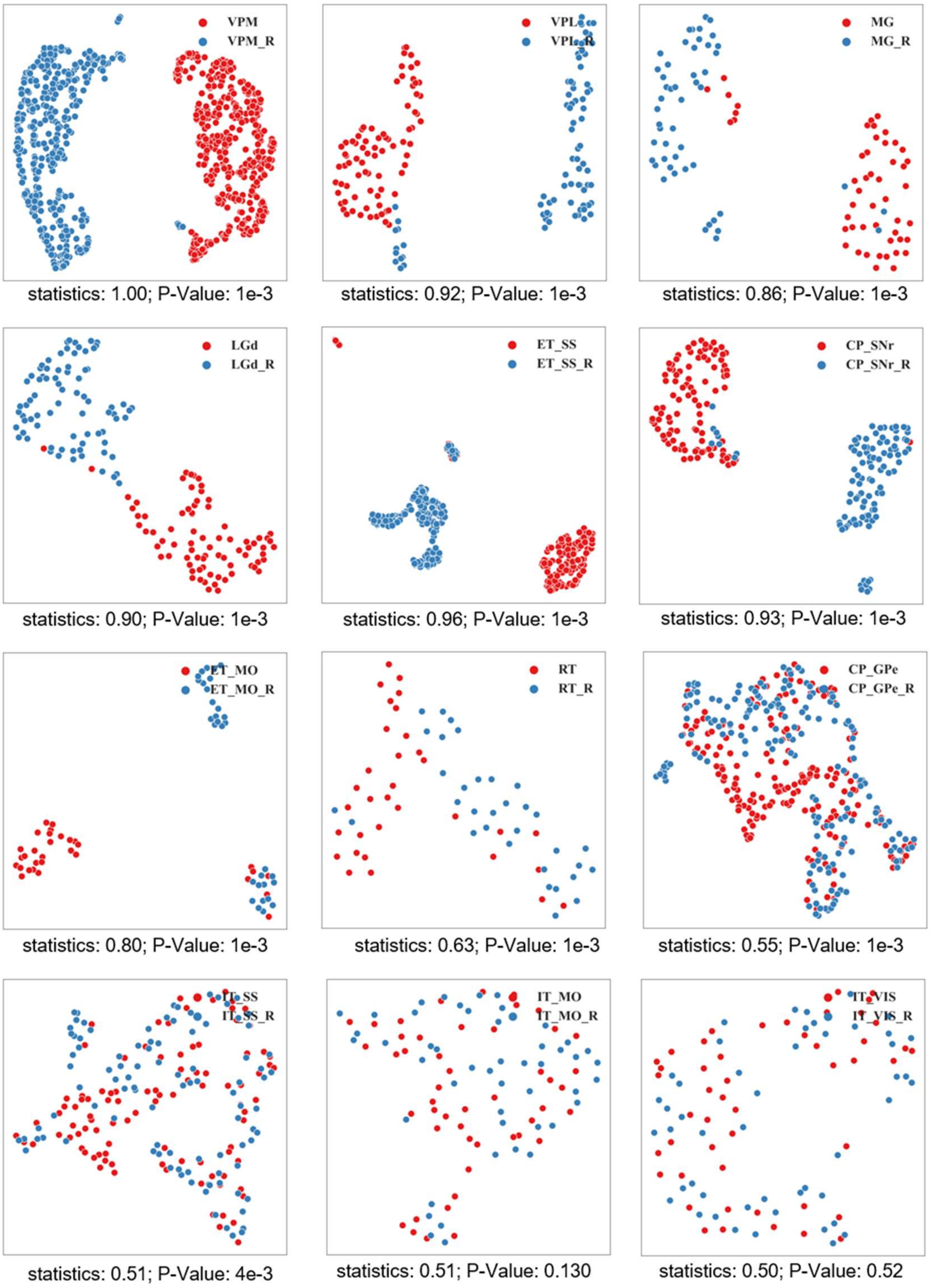

Figure S4. Clustering of HAN neuronal encodings for cells with or without sequence reverse

Each dot represents a neuron and each plot shows the results of UMAP dimension reduction. Here, we tested all the 1,282 cells from twelve cell types (from the SEU dataset) and colored original (red) and reversed (blue) cells. The statistics and P-Values of one-side 'One Sample Discriminability test' are displayed below the plots.

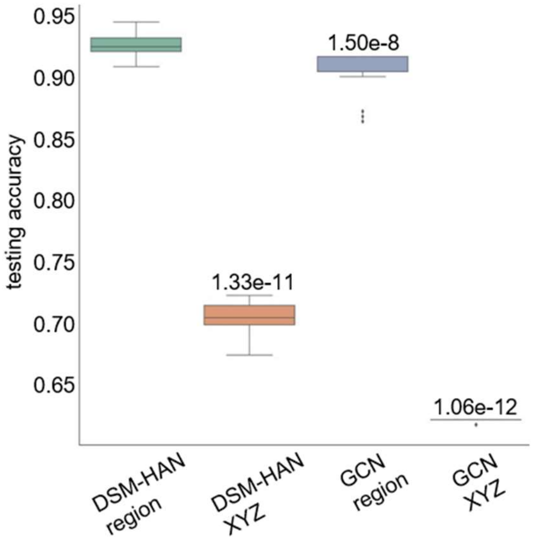

Figure S5. Comparison between brain region features and direct node coordinates

The comparison between our brain region features (W2V encodings), and node coordinates (X-Y-Z) using DSM-HAN on the SEU dataset. Our brain region feature achieved the higher classification accuracy (92.76%), while the other is 71.44% (numbers above box: P-Values of the Mann-Whitney U rank test (one-side) on test accuracy between two features).

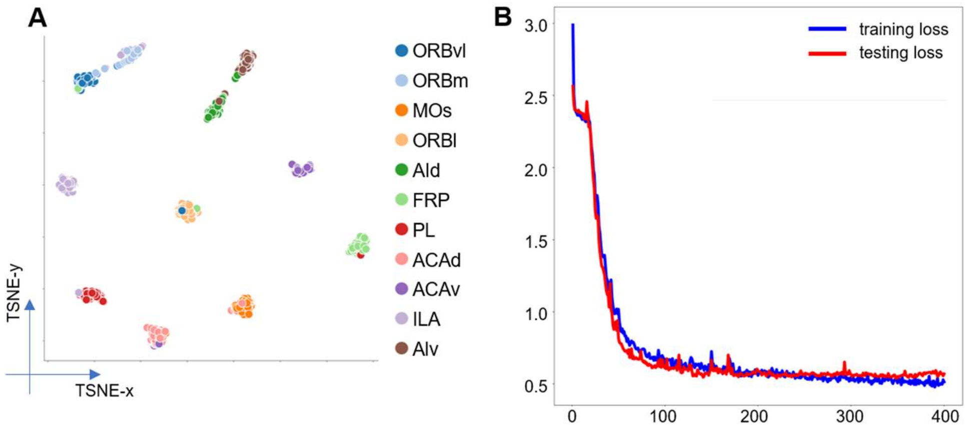

Figure S6. Classification in the 1,100 cells including 11 cell types

A. UMAP embeddings of the 1,100 cells encoded by DSM-HAN (average testing accuracy, 92.2%). B. Training process of DSM-HAN on the new dataset.

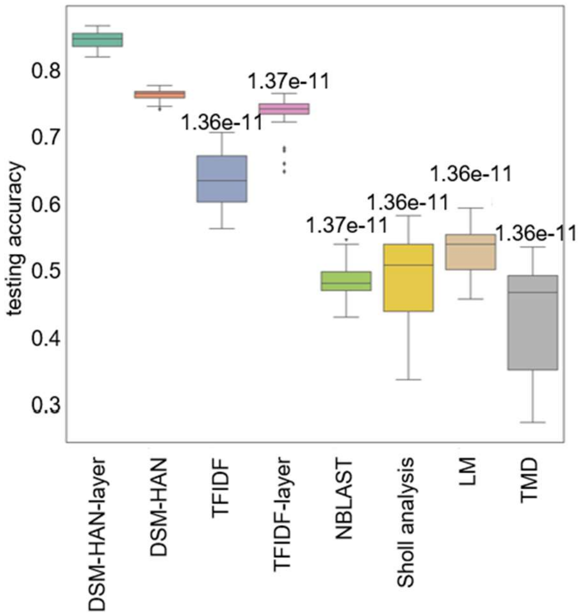

Figure S7. The comparison between DSM-HAN and alternative methods on the local morphology

Local morphologies are dendrites from SEU dataset, and DSM-HAN achieved the highest classification accuracy (76.4%). Accuracy of alternative approaches: TF-IDF = 63.8%, NBLAST = 48.6%, Sholl analysis = 49.5%, L-measure = 53.0%, TMD = 43.0%. We further refined brain region assignment considering layers of brain regions, and received accuracy of DSM-HAN-layer, 84.7%, and TFIDF-layer, 73.6% (numbers above box: P-Values of the Mann-Whitney U rank test (one-side) on test accuracy between DSM-HAN and respective method).

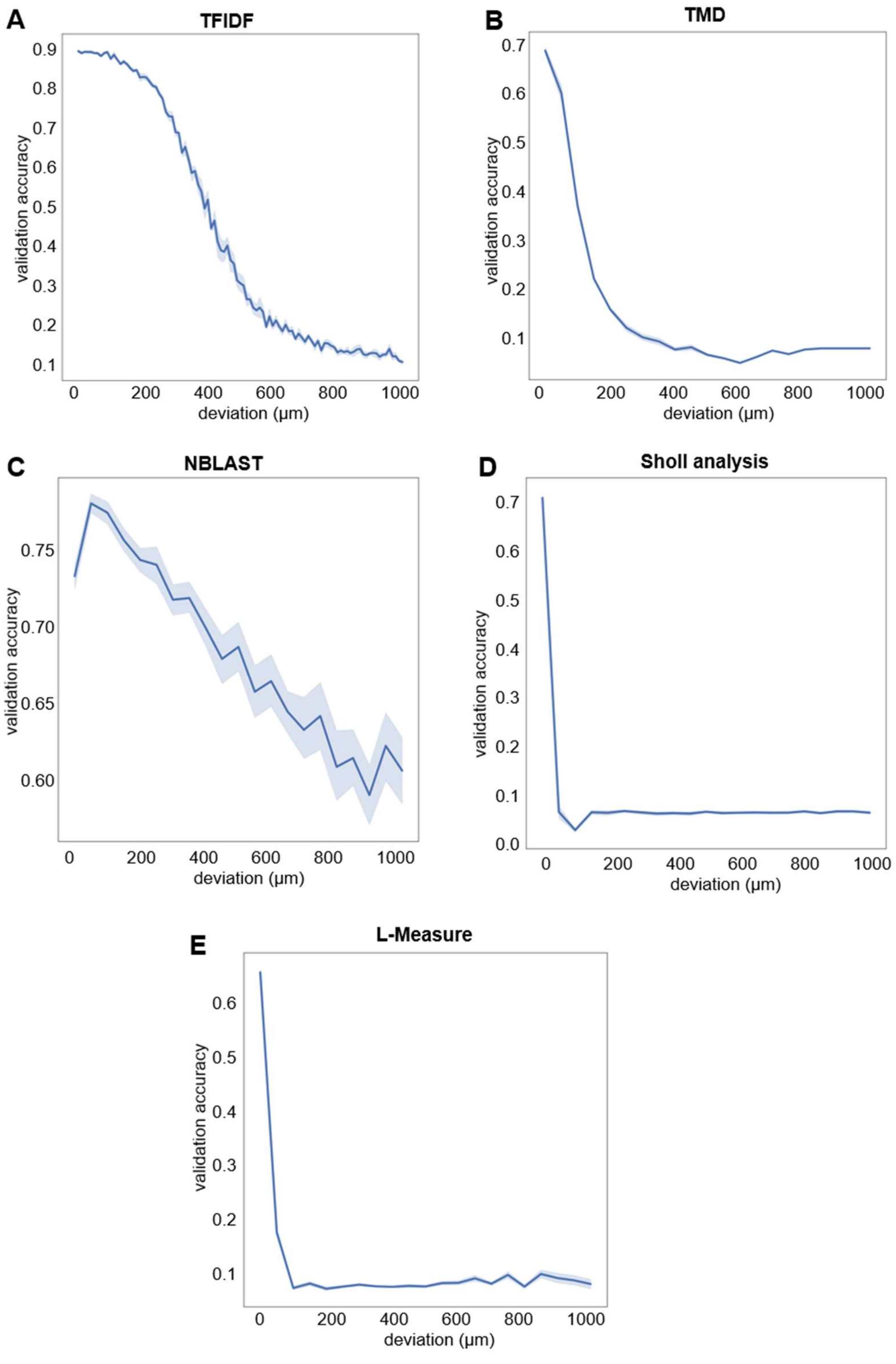

Figure S8. Comparison of robustness for alternative approaches

A-E. The robustness test to spatial noises for alternative approaches (TFIDF, TMD, NBALST, Sholl Analysis, and L-Measure). The Gaussian noises (mean = 0mm) were applied on these neuron reconstruction coordinates, with noise level (the standard deviation of Gaussian distribution) gradually increased from 10mm to 1000mm (step size: 50mm). Testing results indicated that TFIDF and NBLAST were able to maintain their accuracy under spatial noise < 100 mm, while the performance of others fell dramatically.

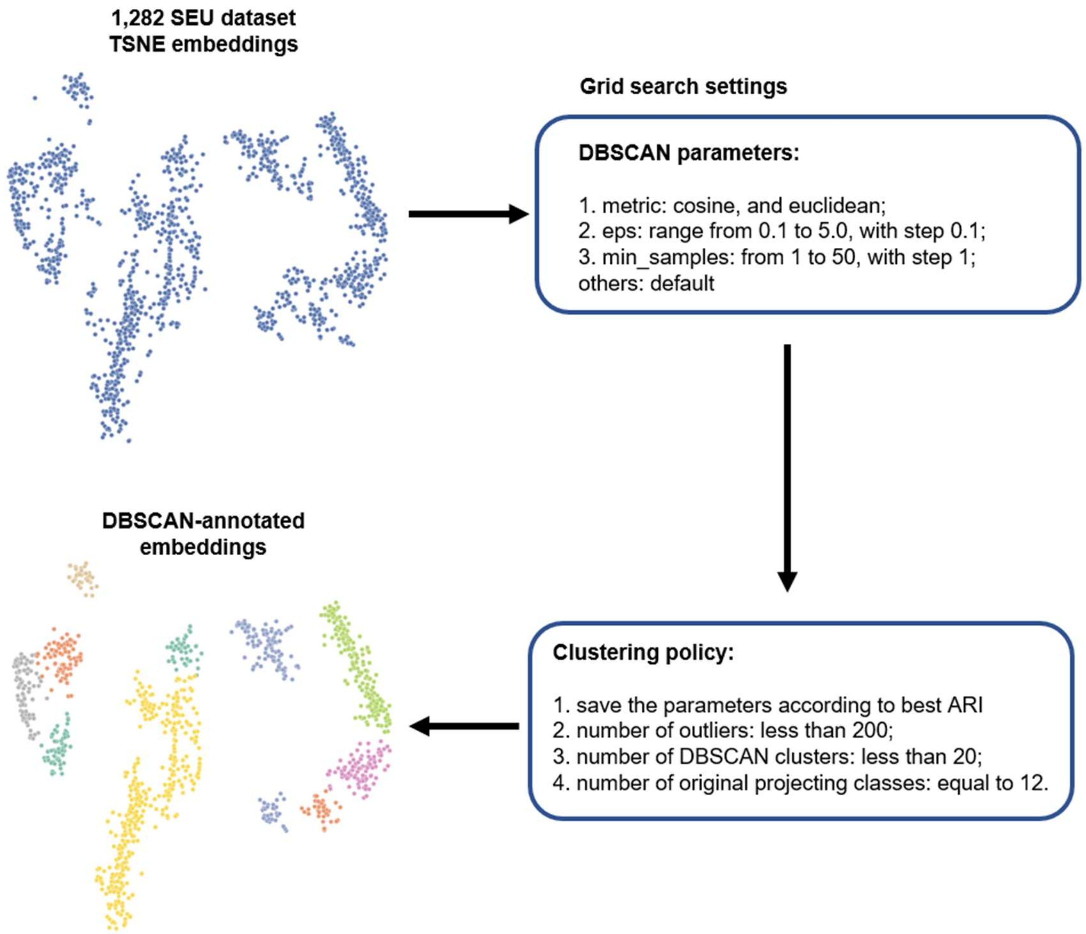

Figure S9. Clustering policy for DBSCAN algorithm

We pre-define parameter space for grid searching, and the input embeddings are passed to DBSCAN for clustering according the policy. The best clustering results were saved for annotating.

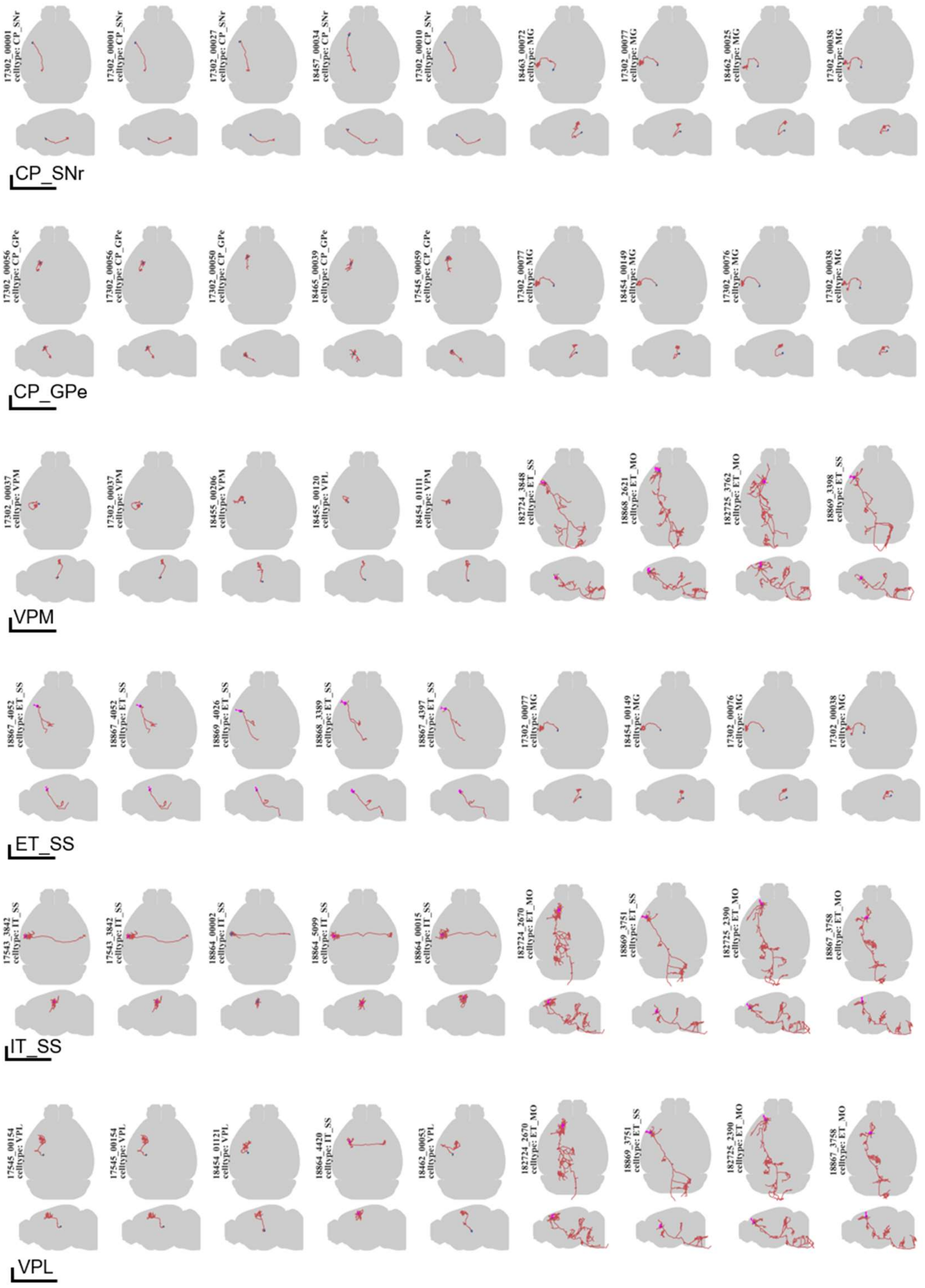

Figure S10. Cell retrieval examples

For the cell retrieval task, we provide both target cells and not-target cells for query cell, illustrating the diversity of neuron morphologies in this dataset and that this is also captured by our method. Columns 1 represents input cell, columns 2-4 represent target cells , and columns 6-9 represent non-target cells.

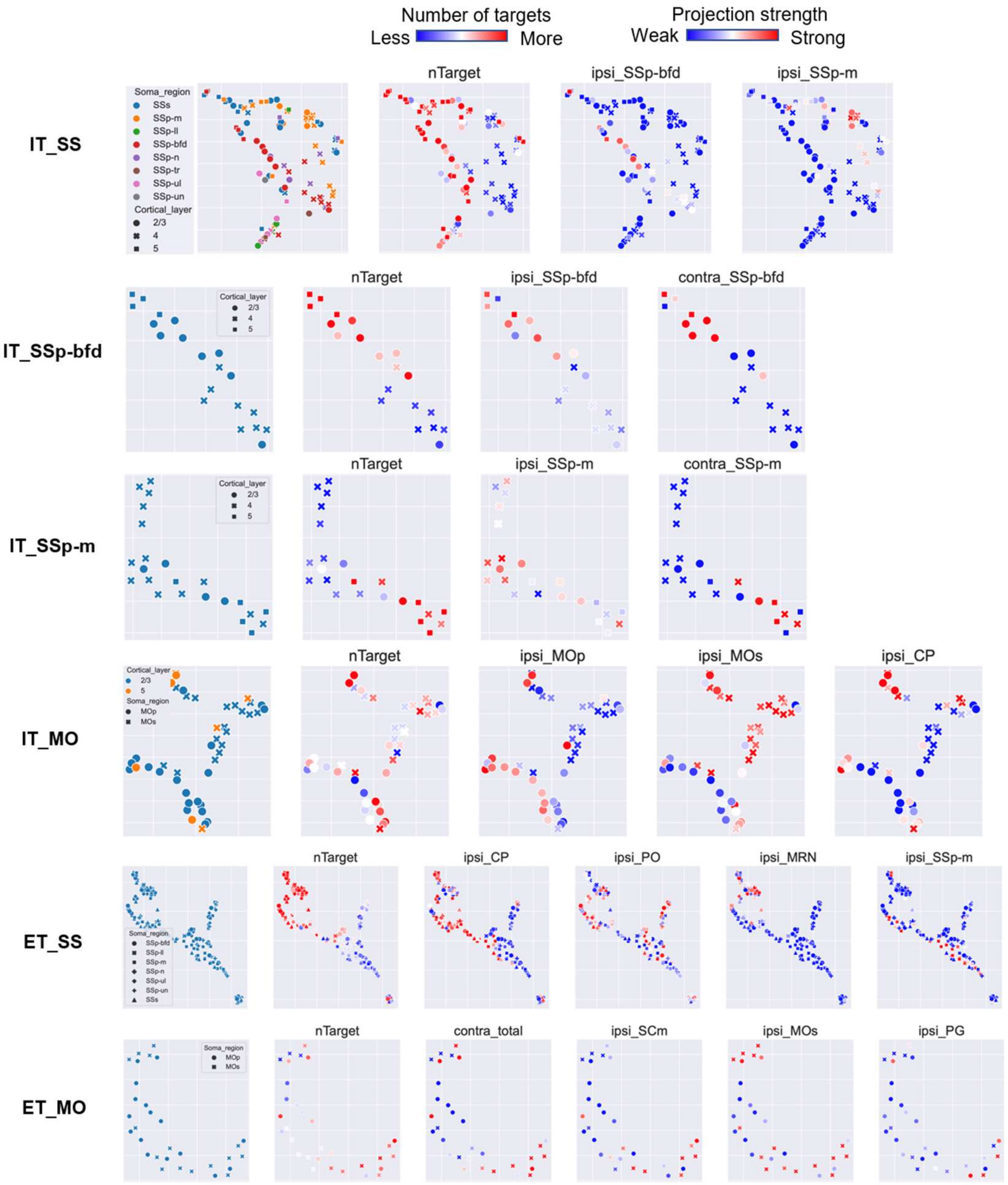

Figure S11. Re-clustering analysis for 6 cell-types of the SEU dataset

Re-clustering was operated on the DSM-AE representation of neurons. For each cell-type, the UMAP layout was shown. Soma region, cortical layers or projection sub-types were indicated by categorical colors or shapes. Number of targets and projection strength of representative target regions were represented gradient colors. For IT_SS cells, re-clustering of two sub-types (IT_SSp-bfd and IT_SSp-m) was shown.

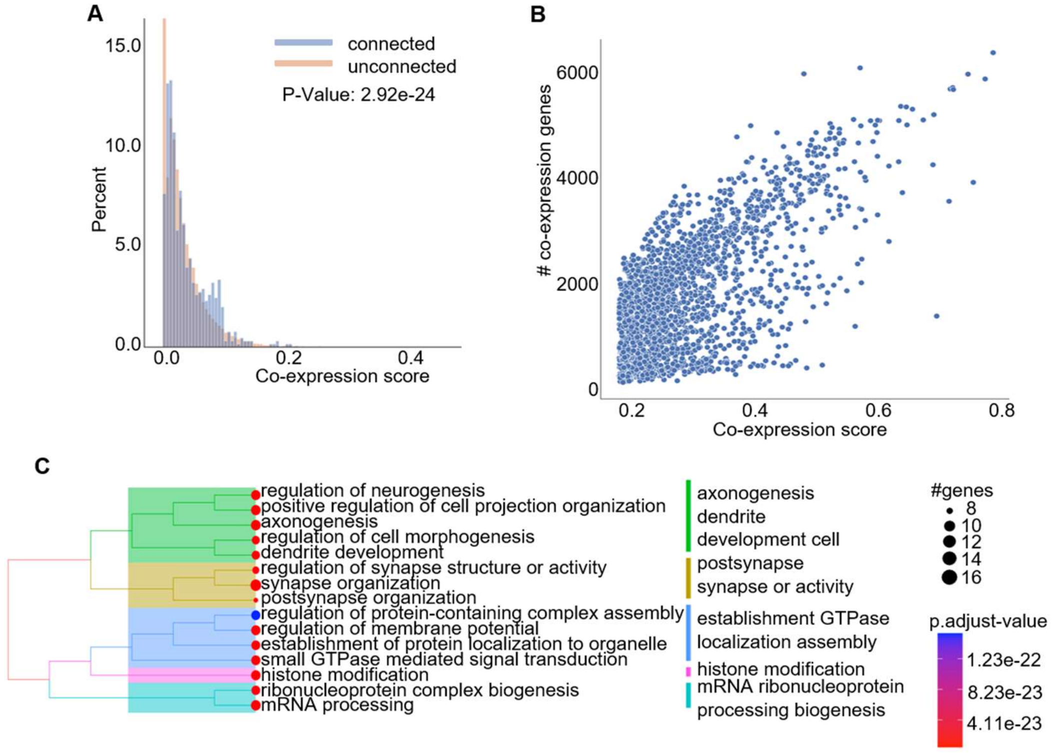

Figure S12. Supplemental co-expression analysis

A. Histogram for the distribution of co-expression scores of connected and unconnected brain regions pairs, which are from different major brain region. 'Percent' indicates the percent of observations that fall within a specific bin. Colors represent brain regions' state (P-Value: 2.92e-24, two-sided KS test). B. Co-expression brain regions. Scatterplot of 2,251 pairs of connected regions with strong co-expression (selected by co-expression score, threshold 0.180). The x- and y-axis represent co-expression scores and the numbers of co-expression genes. C. Functional enrichment analysis of genes expressed in the brain atlas. GO tree-plot results show the clustering of top-15 enriched functional terms (adjusted P-Values, hypergeometric test, 'BH' correction).

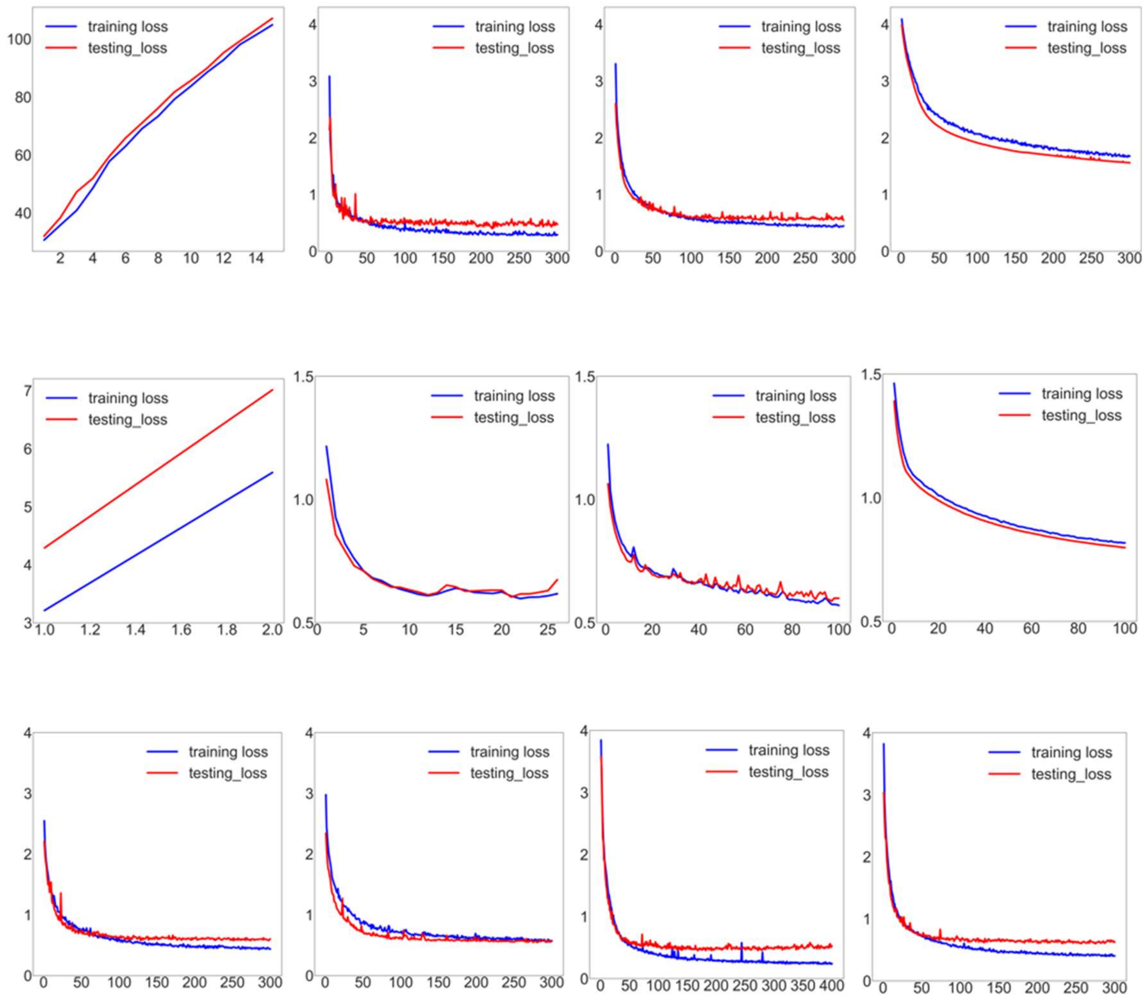

Figure S13. Training details of DSM-HAN

A. Learning rate tests for training of DSM-HAN model. From left to right, learning rate significantly affects the convergence of training. With LR=0.1, the training loss dramatically increased. With LR=0.0001, the rate of convergence was low as the validation loss was still decreasing after 300 training iterations. With LR=0.01 or 0.001, reasonable convergence can be observed. B. Learning rate tests for training of DSM-AE model. C. The classification performance under different hidden layer dimensions of classification network (dim = 16, 32, 64, 128). We found that this parameter can hardly affects the performance.

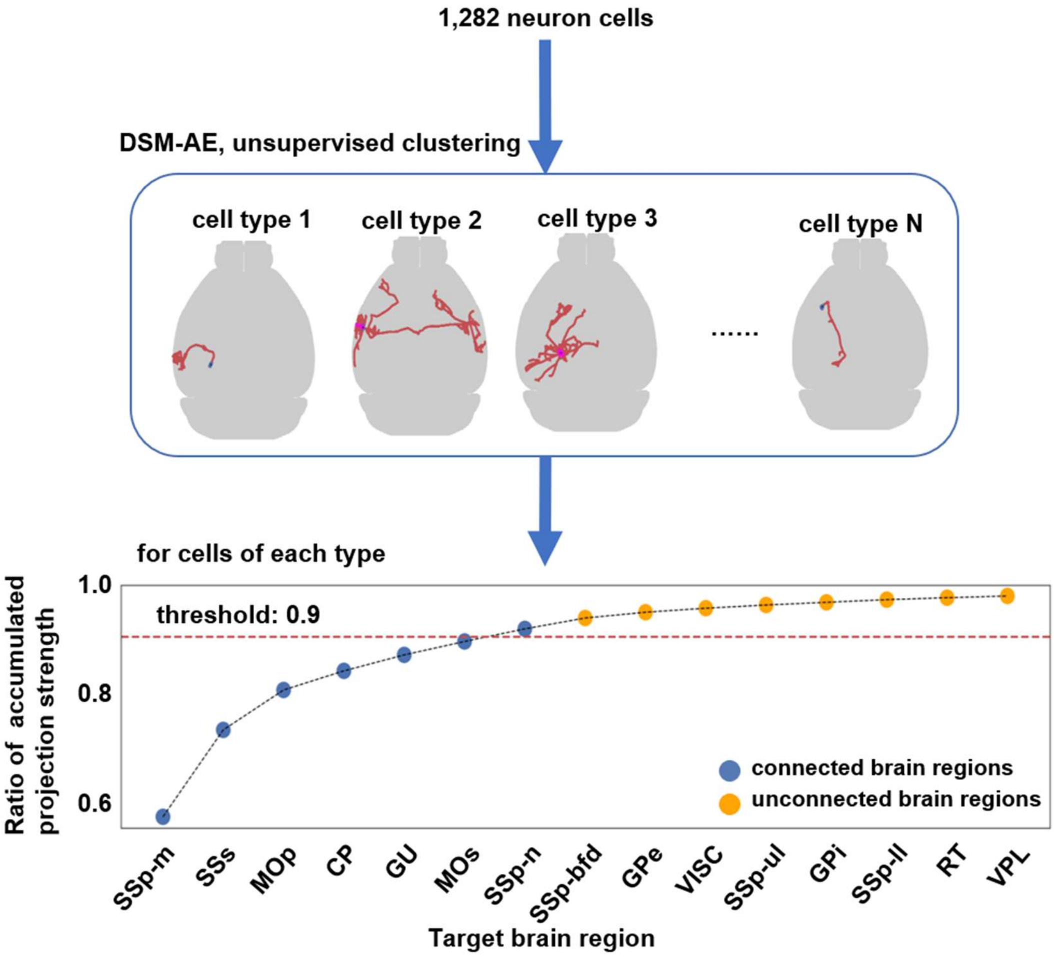

Figure S14. Policy for determining brain region connection

To retrieve the brain region connections at whole mouse brain level, we first identified the 1,282 neuron cells into 11 clusters, using DSM-AE embeddings and DBSCAN algorithm. For each cell type, we reordered their target brain regions in descending order by averaged projection strength (from left to right). By accumulating the first N averaged projection strength until 90% of total projection strength, the first N regions are defined as connected. The regions below blue dots represent connected regions, while the regions below orange dots are unconnected regions.

Supplemental Tables

Table S4. The measurements of ARI and discriminability on DSM-AE representations.
The correspondence of clusters and cell types is evaluated by ARI, and we calculate the ARI metric under alternative post-processing methods. The quantification of DSM-AE representation, is evaluated by discriminability, which also takes post-processing methods into consideration.

	Standardization	PCA	ISOMAP	MDS	LLE	UMAP	TSNE
DSM-AE	**0.577**	**0.549**	**0.605**	**0.544**	**0.592**	**0.677**	**0.719**
TFIDF	0.105	0.252	0.486	0.423	0.408	0.400	0.459
NBLAST	0.107	0.001	0.170	0.006	0.045	0.018	0.207
TMD	0.092	0.065	0.201	0.065	0.451	0.336	0.501
Sholl analysis	0.000	0.001	0.006	0.002	0.007	0.013	0.004
L measure	0.042	0.128	0.525	0.036	0.535	0.620	0.665
flatten neuron sequence	0.144	0.145	0.468	0.002	0.060	0.384	0.553

Table S6. Outlier detector performance.
An outlier detector is trained to filter unknown cells (not belong to the 12 classes). The training is performed on SEU dataset, and the testing is on Janelia dataset.

**Celltype**	**num_cells**	**HAN_prediction**	**outlier_detection_correction**	Note: training dataset comes from seu(1282 cells)
VPM	378	386	356	
VPL	80	73	57	
IT_MO	48	51	51	
ET_MO	31	29	24	
IT_VIS	48	47	39	
ET_SS	159	162	159	
IT_SS	97	95	82	
MG	50	50	36	
CP_GPe	180	180	166	
CP_SNr	100	97	79	
LGd	78	78	72	
RT	33	34	25	
(sum)	1282	1282	1146	

Table S12. Summary of 22 L-Measure features used in this study.

Number of Nodes
Soma Surface
Number of Stems
Number of Bifurcations
Number of Branches
Number of Tips
Overall Width
Overall Height
Overall Depth
Average Diameter
Total Length
Total Surface
Total Volume
Max Euclidean Distance
Max Path Distance
Max Branch Order
Average Contraction
Average Fragmentation
Average Parent-daughter Ratio
Average Bifurcation Angle Local
Average Bifurcation Angle Remote
Hausdorff Dimension
